# Exposure of HepaRG Cells to Sodium Saccharin Underpins the Importance of Including Non-Hepatotoxic Compounds When Investigating Toxicological Modes of Action Using Metabolomics

**DOI:** 10.3390/metabo9110265

**Published:** 2019-11-04

**Authors:** Matthias Cuykx, Charlie Beirnaert, Robim Marcelino Rodrigues, Kris Laukens, Tamara Vanhaecke, Adrian Covaci

**Affiliations:** 1 Toxicological Centre, University of Antwerp, Universiteitsplein 1, 2610 Wilrijk, Belgium; 2 Research Group In Vitro Toxicology and Dermato-Cosmetology (IVTD), Vrije Universiteit Brussel, Laarbeeklaan 103, 1090 Jette, Belgium; Robim.marcelino.rodrigues@vub.be (R.M.R.); Tamara.vanhaecke@vub.be (T.V.); 3 Department of Mathematics & Computer Science, University of Antwerp, Middelheimlaan 1, 2020 Antwerp, Belgium; Charlie.beirnaert@uza.be (C.B.); Kris.laukens@uantwerpen.be (K.L.); 4 Biomedical Informatics Network Antwerpen (Biomina), University of Antwerp, Middelheimlaan 1, 2020 Antwerp, Belgium

**Keywords:** in vitro, HepaRG, sodium saccharin, reference toxicants

## Abstract

Metabolites represent the most downstream information of the cellular organisation. Hence, metabolomics experiments are extremely valuable to unravel the endogenous pathways involved in a toxicological mode of action. However, every external stimulus can introduce alterations in the cell homeostasis, thereby obscuring the involved endogenous pathways, biasing the interpretation of the results. Here we report on sodium saccharin, which is considered to be not hepatotoxic and therefore can serve as a reference compound to detect metabolic alterations that are not related to liver toxicity. Exposure of HepaRG cells to high levels of sodium saccharin (>10 mM) induced cell death, probably due to an increase in the osmotic pressure. Yet, a low number (*n* = 15) of significantly altered metabolites were also observed in the lipidome, including a slight decrease in phospholipids and an increase in triacylglycerols, upon daily exposure to 5 mM sodium saccharin for 72 h. The observation that a non-hepatotoxic compound can affect the metabolome underpins the importance of correct experimental design and data interpretation when investigating toxicological modes of action via metabolomics.

## 1. Introduction

Since the introduction of metabolomics as a new “-omics” domain in 1999, the field of research has proved to be a valuable source of information for the actual phenotype or state of organisms [1]. Metabolomics is defined as the study of the biochemical profile of small molecules in an organism [1]. Because the metabolome is the most downstream level in the biomolecular organisation of a system, metabolomics fingerprints are very dynamic and alterations may be induced even by small external triggers [2,3]. Many of these stimuli, such as gender, age (young vs. old), dietary status (e.g., fasting vs. fed) and activity (rested vs. active) potentially form biases that obscure the metabolic signature related to exposure. These biases are important, but can be anticipated through strict subject selection criteria and proper randomisation [4].

An effect often not considered is the exposure itself. Although this placebo effect is well-known in medicine, precautions are often not taken during metabolomics studies. The “exposure bias” is relevant when combining metabolomics with in vitro experiments, since it is one of the main potential sources of bias. Although the biological variation and bias is reduced in cell culture experiments, confounding factors still pose a risk during metabolomics investigations. Indeed, the mere presence of a xenobiotic may theoretically cause a metabolic shift, even though this compound is not considered harmful. This confounding factor of “exposure” can generate results falsely interpreted to be related to toxicity [5].

Sodium saccharin is an artificial sweetener that has been used for over a century. Except for a case study describing an idiosyncratic reaction after exposure to different pharmaceutical products containing the sweetener, no evidence of human hepatotoxicity has been reported so far [6,7]. The safe characteristics of sodium saccharin make it a good candidate to investigate potential metabolic alterations triggered in vitro upon exposure to a non-hepatotoxic molecule.

## 2. Materials and Methods

### 2.1. Materials and Methods

Materials, exposure and acquisition methods have been performed as described previously [8]. A brief description highlighting the principles is mentioned in-text and full details concerning the experimental protocols are provided in the Supplementary Materials SM-1 to SM-5.

### 2.2. Determination of Testing Concentrations

Seven days after initial cell seeding, the wells were divided into two negative control groups and eight groups that were exposed to sodium saccharin at different concentrations ranging from 0.40 to 40 mM for a period of 72 h in a repeated dose exposure, in which the medium was refreshed every 24 h. Viability was assessed using the neutral red uptake (NRU) assay [9,10]. Full details are available in the Supplementary Materials SM-2.

### 2.3. Metabolomics Experiments

#### 2.3.1. Seeding of the HepaRG^®^ Cells and Exposure to Sodium Saccharin

Cryopreserved differentiated HepaRG^®^ cells were thawed and seeded in collagen-coated two-well Lab-Tek chamber slides at a density of 1.03 × 10^6^ cells/well. Two additional blank chamber slides were treated identically to serve as blanks during further analysis. After seven days of cultivation, the cell cultures were visually checked for hepatocyte/biliary cell ratio and block randomised in three groups: a negative control group in comparison to a dose of sodium saccharin at a concentration of 5.5 mM (high dose) and a 1/10 dilution of the high dose, i.e., 0.5 mM (low dose). Higher concentrations of sodium saccharin were not applied because of hyperosmotic toxic effects (additional osmotic pressure >20 mOsm/L). Each group contained six replicates, which is often considered an adequate sample size for in vitro metabolomics experiments [5,11]. The cell cultures were exposed for 72 h with a medium refreshment every 24 h. The exposure experiment has been performed twice to reduce false positive results [8].

#### 2.3.2. Sample Preparation

The cell cultures were harvested according to previously described protocols, full details are available in SM-3 [12,13]. Briefly, cells were prepared for extraction with a wash in phosphate buffered saline (37 °C) followed by freezing on liquid nitrogen. Cells were scraped from the surface three times with 200 µL of a cooled (−80 °C) 80% (v/v) methanol (MeOH)/milliQ water solution. Liquid/liquid extraction was performed using ultrapure water, methanol, and chloroform. Quality control (QC) pools were generated through the collection of aliquots of all samples for the polar and non-polar phases [14]. Both fractions were evaporated to dryness and reconstituted in LC-MS-compatible solvents.

#### 2.3.3. LC-MS Analysis

LC-MS analysis was performed using separation mechanisms described in SM-4 [8,15]. The non-polar fraction was analysed using reversed phase chromatography on a Kinetex XB-C18 (150 × 2.1 mm; 1.7 µm particle size, Phenomenex, Utrecht, the Netherlands). Mobile phase compositions were mixtures of methanol, isopropanol (IPA) and water with ammonium acetate (pH 6.7) and of acetonitrile (ACN), IPA and water with an acetate buffer (pH 4.2) for negative and positive ionisation modes, respectively.

The polar fractions were analysed using HILIC systems using an iHILIC column (100 × 2.1 mm; 1.8 µm particle size, HILICON, Umea, Sweden) with ACN, MeOH and water with an ammonium formate buffer (pH 3.15) for the positive ionisation mode, and a polymeric iHILIC Fusion Column (100 × 2.1 mm, 5 µm particle size, HILICON) in combination with ACN, MeOH and water with an ammonium carbonate ((NH_4_)_2_CO_3_) buffer (pH 9.0) for the negative ionisation mode. LC-separation was performed on an Agilent Infinity 1290 UPLC (Agilent Technologies, Santa Clara, CA, USA), connected to an Agilent 6530 QTOF with Agilent Jet Stream nebuliser (Agilent Technologies). The LC-MS system was equilibrated using 15 QC-injections at the start of the data acquisition. The injection order of the samples was block-randomised to prevent bias related to instrumental drift. One QC injection was performed after every four sample injections to monitor instrumental drifts.

#### 2.3.4. Data Analysis

##### Data Quality Control

Internal standards were used to evaluate the precision of the retention time and m/z-accuracy within and between experimental batches. The raw data were searched for the internal standards using the Find by Formula algorithm (Agilent Technologies) with the following parameters: formula matching ± 10 ppm, expected variation 2 mDa ± 8 ppm. Samples were only considered when internal standards were detected and the number of molecular features was comparable to those of the other samples. The absence of internal standards and/or molecular features in an acquired LC-MS run may indicate analytical issues during the run and, therefore, the removal of the failed runs was considered to improve the quality of the final dataset. The results of internal standard quality control were used to set the parameters for further data processing.

##### Data Pretreatment

Acquired data were imported on the MassHunter Qualitative software (Agilent Technologies, v 2.06.00) and converted to centroid m/z data. The generic datafiles were processed using the XCMS package in the R workspace [16,17]. Features representing the ions of the extracted metabolites were searched using the centWave algorithm. Features were aligned with the Obiwarp algorithm and grouped by density [18]. Missing peaks were re-extracted using the fillPeaks algorithm [17].

The dataset was cleaned by removing isotopes and features present in blank samples. Other applied filters were based on a high number of missing values and within-group variability. All preprocessing functions were executed using the MetaboMeeseeks package [19]. A principal component analysis (PCA) was performed and outliers were removed for further analysis (*n* = 4) (Figure S6). After outlier removal, the filter process was re-iterated and samples were normalised using BatchCorr normalisation [20]. Missing values imputation was considered but not applied, since it had no positive impact on the within-group variance. The final dataset was once more evaluated using a PCA to assess important trends and their potential impact on the subsequent multivariate analysis. All parameters of feature extraction and data clean-up are mentioned in SM-5.

##### Statistical Analysis

Univariate statistical analysis was performed through the non-parametric Mann–Whitney U test with a Benjamini–Hochberg correction for multiple testing using the multtest package in R [21]. In addition, a partial least squares discriminant analysis (PLS-DA) and a random forest classification were performed as multivariate analyses [19,22]. Performances were checked using leave-one-out cross-validation. Metabolic alterations were defined based on significance in the univariate tests (*q*-value <0.05) and on importance in the multivariate models (based on the covariance of the latent values of the first component of the PLS-DA and the bimodal distribution of the variable importance measure (VIM) of the random forest classifier model). The raw signals of the selected signals were manually checked to confirm the result.

##### Metabolite Annotation

The details of metabolite annotation are mentioned in SM-6. Briefly, annotation was performed in Mass Hunter using the molecular feature extractor algorithm: the signals corresponding to the altered metabolite were selected, the complete result set was extracted and the Molecular Formula Generator (MFG) generated a list of possible chemical formulas. The identification was based on the m/z-value, the isotope pattern, the measured retention time and the fragmentation spectra acquired during the equilibration runs. Results were reported according to the standards of the CAWG and MSI [23,24]. Level 2 and level 3 identifications were considered of sufficient quality to infer a biological interpretation to the outcome of the experiments. All metabolites, including molecular features with lower levels of confidence in annotation (levels 4–5), are reported in Table S4.

## 3. Results

### 3.1. Experimental Observations

**Figure 1:**
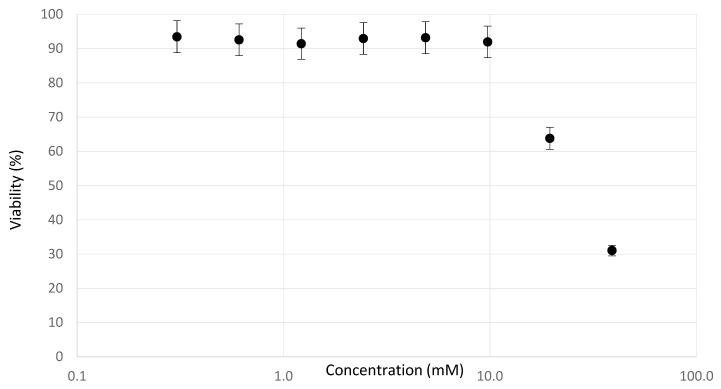
Averaged viability curve for the neutral red uptake (NRU)-assay upon daily exposure to sodium saccharin for a period of 72 h. Cytotoxicity is observed from 10 mM onwards, which is equal to an osmotic pressure of ±20 mOsm/L.

The dose–response curves of the viability assay in Figure 1 showed a clear decrease in viability from 10 mM sodium saccharin onwards. Indeed, concentrations of 10 mM increased the osmotic pressure over 20 mOsm/L to supra-physiological ranges [25]. Hyperosmolarity is a form of toxicity not related to physiological hepatotoxicity, and such high exposures are therefore not considered as a good reference for investigating the chemical hazard of the product. A high-dose exposure of 5 mM induced an osmotic pressure of ±10 mOsm/L, yet no cytotoxic effects were observed.

### 3.2. Data Quality

The injections that were not considered during statistical analysis are reported in Table S2. For the non-polar fraction, injections QC-1 and QC-2 were excluded from the analysis because of failed injections, reflected by the absence of internal standards in the chromatogram and the absence of the typical chromatogram.

As shown in Table S3, standard deviations were higher during the first experimental batch due to autosampler thermostatic issues. The high mRSDs for QCs in comparison to all other experiments using lipidomics approaches can be explained by the shift in retention times between the two experimental batches. To correctly match corresponding peaks, the parameters for alignment and grouping were less strict, which introduced extra noise in the data.

### 3.3. Selection of Potential Endogenous Markers of Exposure

**Figure 2:**
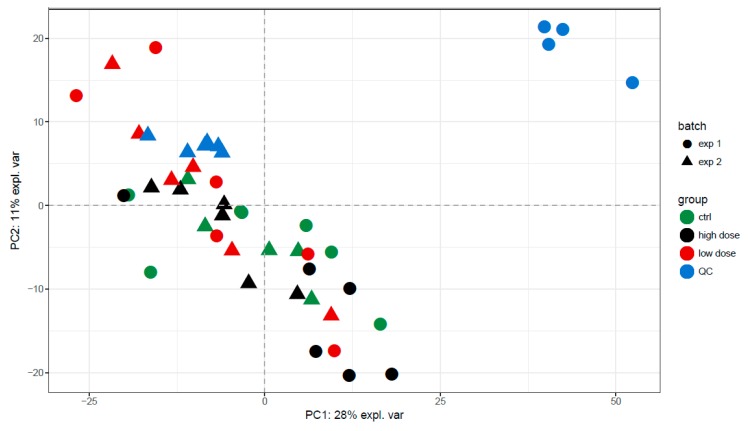
PCA plots of the non-polar fraction in positive mode during the 72-h exposure showing PC1 vs. PC2. There is strong overlapping of the different exposure groups in all principal components, indicating the variance is not related to exposure. Only a slight trend is visible between the negative control group and the group exposed to the higher dose.

As shown in the PCA plots in Figure 2 and Figures S7–S10, no clear distinction between all exposure groups was observed. The overlap between the different exposure groups indicated that the source of variation in the dataset was probably not related to the exposure. This was also reflected by the poor performance of both multivariate and univariate tests: all AUCs for the random forest classifiers reported in Table 1 were below 0.7, except for the high-dose exposure conditions. According to the R^2^ and Q^2^ values in Table 2, the PLS-models overfit, with Q^2^ values < 0.2. The R^2^ of the non-polar fraction in positive mode was good (>0.8), but the cross-validation showed this was an overfit value, and the Q^2^ was reduced to 0.40.

Univariate tests did not reveal major differences between the exposure groups and the negative control group. Only 15 features were observed to be significantly different between the negative control group and the high-dose exposure group, and only for the non-polar fraction in positive mode.

The identified features given in Table S4 represent a decreased presence of six phosphatidylethanolamines, two phosphatidylinositols and one sphingomyelin. Further differences included the increase of two low-saturated triacylglycerols. Their respective boxplots are represented in Figure S10.

## 4. Discussion

The metabolome is a dynamic level of the cellular organisation. External stimuli theoretically invoke a response of the cell, resulting in a change of the metabolome. Sodium saccharin is considered to be a non-hepatotoxic chemical, which makes it an ideal compound to select markers of exposure not necessarily related to hepatotoxicity [8,9].

The acquisition of the metabolome of cells exposed to a hepatotoxicant in comparison to vehicle only would reveal alterations that would all be addressed to a toxic mode of action. The main consequence of this assumption is the questionable predictive value of the observed metabolic alterations, especially in small-scale experiments (one dose, one time point exposure of a single chemical exposure). This consideration stresses the importance of an exposure to a non-toxic negative control during the experiment.

Few features of the lipidome were changed significantly (*n* = 15) and included the presence of triacylglycerols and a lower presence of phospholipids. The absence of significant changes in the polar fraction suggested that the cell culture did not implement major adaptations in metabolism as a response to the external stimuli.

Effects related to hyperosmolarity have also been described in human cell cultures, which showed the increased presence of monosaccharides and amino acids to retain the osmotic balance [26]. However, it is possible that these effects were not observed in this current experiment due to the deliberate choice to avoid these hyperosmolar (non-physiological) concentrations. The choice of a supra-physiological dose would imply a bias to select metabolic alterations related to modes of action not relevant in physiological conditions. This bias is especially relevant in in vitro techniques, as exposure concentrations can be increased to unrealistic, non-physiological levels. A potential prevention is the use of toxicokinetic data to confirm the plausibility of the exposure conditions.

García-Cañaveras et al. [27] and Ramirez et al. [28] classified different toxicants according to the mode of action, observing specific fingerprints for different end-points of toxicity. Ramirez et al. described the downregulation of carnitine, creatine, phosphocreatine, and pantothenic acid during exposure to peroxisome proliferating agents, the decrease of oleic acid, galactose and acetyl aspartate in combination with an increase of tryptophan and alanine during exposure to enzyme-inducing xenobiotics [28]. Garcia et al. compared the fingerprint of xenobiotics inducing oxidative stress, steatosis and phospholipidosis, in which they observed alterations in glutamate levels, oxido-reductive status, lysophospholipid/phospholipid ratio and lipid accumulation [27]. The inclusion of non-hepatotoxic compounds in their experimental design, such as citrate and ketotifen, states the importance of reference compounds for non-hepatotoxicity as metabolic changes were observed, albeit with a different fingerprint.

A qualitative comparison of the metabolic alterations for steatosis and cholestasis obtained in previous experiments are presented in Table 3 [8,29]. The clear alterations of the metabolome during hepatotoxic modes of action can be discriminated from the exposure to a non-hepatotoxic toxicant. Next to significant differences in the polar metabolome, the lipidome of a cell culture exposed to hepatotoxic compounds showed clear and strong alterations, with multiple lipid species of several classes involved in the downstream effect, whereas the effects during exposure to sodium saccharin were not substantial.

A mechanistic interpretation based on the observed metabolic alterations provides an additional value to the localisation of the specific molecular initiating event and the potential adverse outcome, as this observation is an additional argument for a toxic cascade. Although metabolic alterations were observed upon exposure to sodium saccharin, no specific affected pathway could be identified. Based on all observations, the use of a non-hepatotoxic compound instead of a vehicle-only negative control group may help reducing false positive results without jeopardising the sensitivity of toxic insults.

## 5. Conclusions

The exposure of HepaRG cells to high levels of sodium saccharin induced cell death, possibly due to osmotic pressure. The exposure concentration should not solely be determined from viability curves, but should also be checked for physiological relevance to prevent unrealistic exposure scenarios. Although sodium saccharin is not considered to be a hepatotoxicant, minor changes were observed in the lipidome, including a slight decrease in phospholipids and an increase in saturated triacylglycerols. The metabolome was altered upon exposure to non-hepatotoxic compounds, indicating the importance of reference compounds when investigating toxicological insults. The metabolic changes were less pronounced than those of reference hepatotoxicants and can therefore be used as a background response to prevent false positive results related to the exposure bias.

## Figures and Tables

**Table 1:** AUCs of the random forest classifiers comparing the negative control group against the different exposures.

Exposure	Non-Polar Positive	Non-Polar Negative	Polar Positive	Polar Negative
Low Dose	0.51	0.55	0.59	0.17
High Dose	0.95	0.40	0.69	0.21

**Table 2:** R^2^ and Q^2^ values for the PLS-DA discrimination between the exposure groups and the negative control group.

Exposure	Non-Polar Positive		Non-Polar Negative		Polar Positive		Polar Negative	
	R^2^	Q^2^	R^2^	Q^2^	R^2^	Q^2^	R^2^	Q^2^
Low Dose	0.01	0.01	0.1	0.06	0.22	0.14	0.21	0.05
High Dose	0.84	0.4	0.42	0.10	0.08	0.03	0.33	0.04

**Table:** (**A**)

	Bosentan				Sodium Valproate				Sodium Saccharin
Time Frame	24 h		72 h		24 h		72 h		72 h
Concentration (µg/mL)	23	230	9.5	95	230	2300	66.5	665	1000
Acetylcholine									
Acetylspermidine									
Aminergic Oligopeptides									
Carnitine									
citric-acid N-sugar									
Choline									
Cholesterol Sulfate									
Creatine									
diacetylspermidine									
GTP									
Isoputreanine									
Methylbutyryl Carnitine									
Methylhydroxylysine									
Nucleotides									
Ornithine									
Pantothenic Acid									
Phosphocholine									
Phosphorylated Metabolites									
Phosphorylethanolamine									
Putrescine									
SAM									
Spermidine									
Taurine									
Trimethylammonium Butanoic Acid									
UDP Glucuronic Acid									
Bile Acids									
Ceramide									
Ceramide, Derivative									
Diacylglycerol									
Glycosfingolipid									
LPE 18:1									
PC									
PE (non PUFA)									
PE (PUFA)									
PE (P)									
PS									
Sfingomyelin									
Triacylglycerol (O)									
Triacylglycerol (>50, PUFA)									
Triacylglycerol (>50, non PUFA)									
Triacylglycerol (<50)									

Abbreviations: GTP, guanosyl triphosphate; LPE, lysophosphatidylethanolamine; PC, phosphatidylcholine; PE, phosphatidylethanolamine; PI, phosphaditylinositol; PS, phosphatidylserine; SAM, S-adenosyl methionine; TG, triacylglycerol.

**Table:** (**B**)

Colour						
Number of Lipid Species	>3	1–3	0	1–3	4–10	>10
Signal Abundance	Lower	Lower	N/A	Higher	Higher	Higher

## Supplementary Materials

The following are available online at https://www.mdpi.com/2218-1989/9/11/265/s1, Figure S1: Viability curves for the NRU-assay 72 h of exposure., Figure S2: Extracted ion chromatogram for all samples in experiment 1 for *m*/*z* 162.1139 at a retention time of 8.7. Figure S3: Boxplots for *m*/*z* 162 at rt 8.7 min. Figure S4: Annotation of the molecular formula using the Agilent Molecular Formula Generator reveals a match for the proton, sodium and potassium adduct of a molecule with formula C_7_H_15_NO_3_, Figure S5: MS/MS spectrum of the molecular feature (up), which matches with the MS/MS spectrum of L-carnitine in the METLIN database (down), Figure S6: PCA reflecting the outlier position of QC-1 and IC10-6 for the dataset of the non-polar fraction in negative ionisation mode, Figure S7: PCA plots of the non-polar fraction in negative mode during the 72 h exposure showing PC1 vs. PC2 (upper) and PC3 vs. PC4 (lower), Figure S8: PCA plots of the non-polar fraction in positive mode during the 72 h exposure showing PC1 vs. PC2 (upper) and PC3 vs. PC4 (lower), Figure S9: PCA plots of the polar fraction in negative mode during the 72 h exposure PC1 vs. PC2 (upper) and PC3 vs. PC4 (lower), Figure S10: PCA plots of the polar fraction in positive mode during the 72 h exposure showing PC1 vs. PC2 (upper) and PC3 vs. PC4 (lower), Table S1: Concentration range (in μg/mL) for the NRU-assay of sodium saccharin on HepaRG cells for a period of 72 h, Table S2: Samples which did not meet QC criteria., Table S3: Median relative standard deviations (mRSDs) for all subgroups in the experiments, Table S4: AUCs of the random forest classifiers comparing the negative control group against the different exposures, Table S5: R^2^ and Q^2^ values for the PLS-DA discrimination between the exposure groups and the negative control group, Table S6: Metabolites identified as potential metabolites of interest for all exposure models.
