# Supplementary material for: Exposure of HepaRG Cells to Sodium Saccharin Underpins the Importance of Including Non-Hepatotoxic Compounds When Investigating Toxicological Modes of Action Using Metabolomics
Source: Metabolites. 2019 Nov 4;9(11):265. doi: 10.3390/metabo9110265 (PMC6918164; doi:10.3390/metabo9110265)
Supplement: Supplementary file 1 [file metabolites-09-00265-s001.zip › supplementary materials/SI 10.pdf]

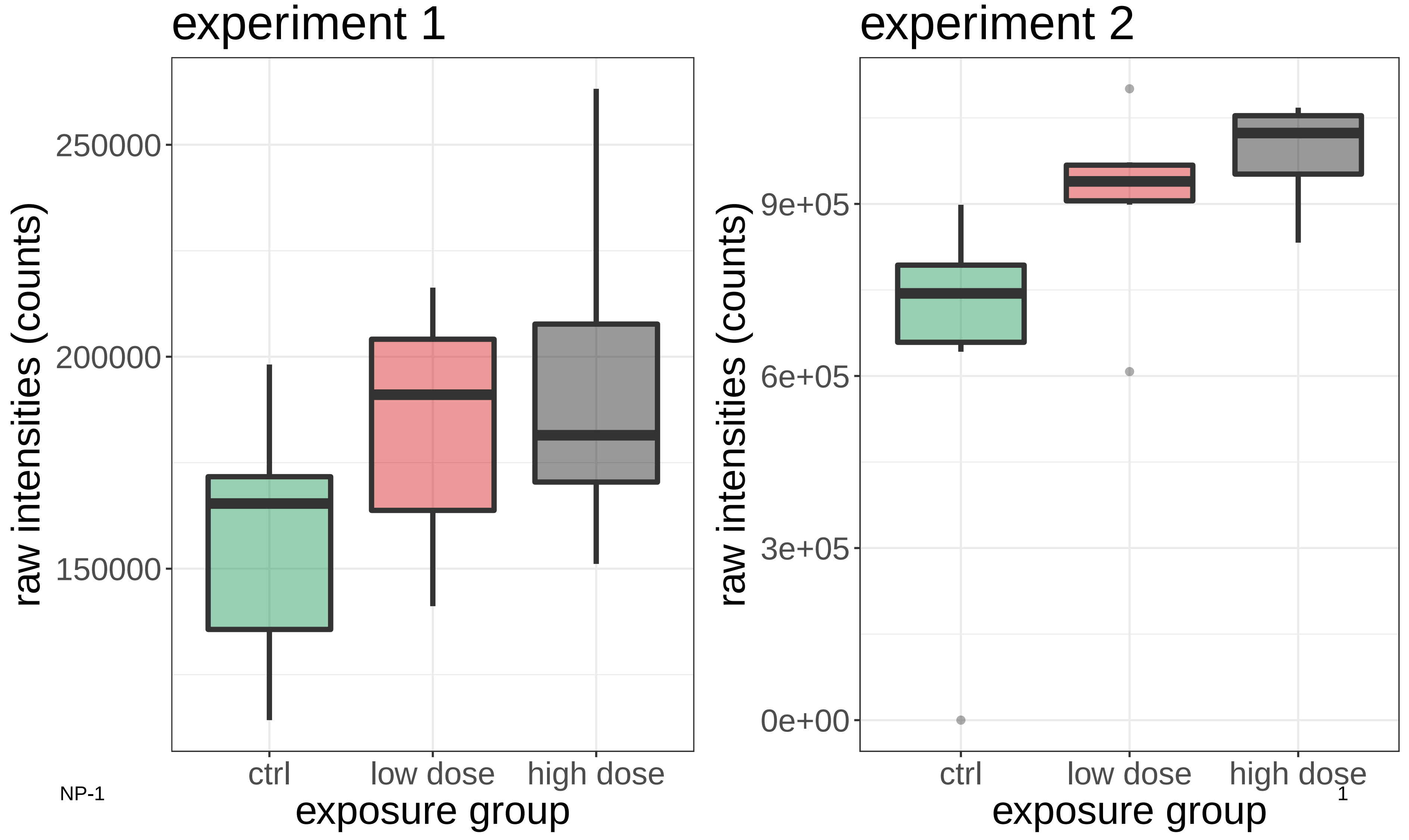
experiment 1
raw intensities (counts)
250000
200000
150000
ctrl
low dose
high dose
exposure group
experiment 2
raw intensities (counts)
9e+05
6e+05
3e+05
0e+00
ctrl
low dose
high dose
exposure group

NP-1

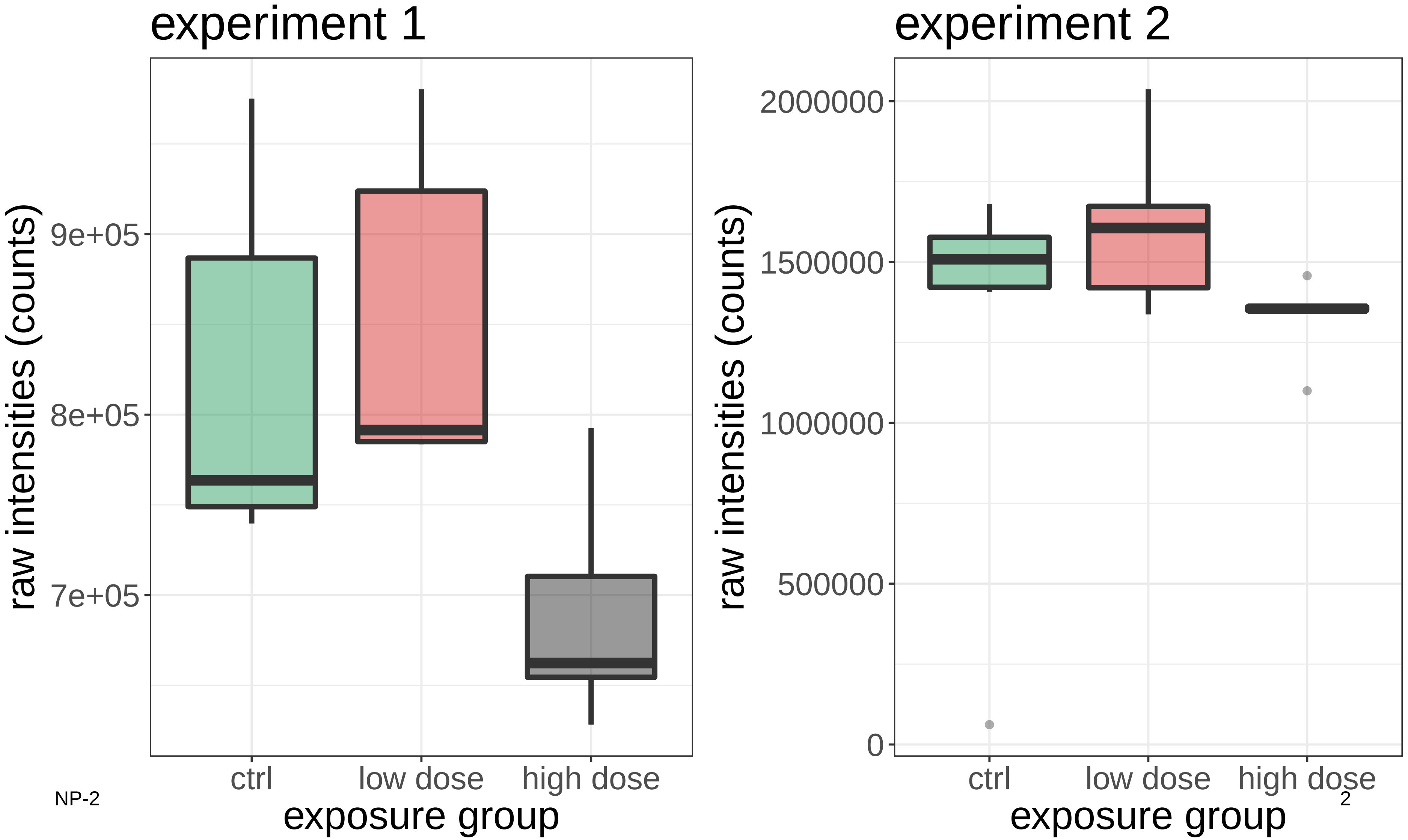
experiment 1
raw intensities (counts)
9e+05
8e+05
7e+05
ctrl
low dose
high dose
exposure group
experiment 2
raw intensities (counts)
2000000
1500000
1000000
500000
0
ctrl
low dose
high dose
exposure group

NP-2

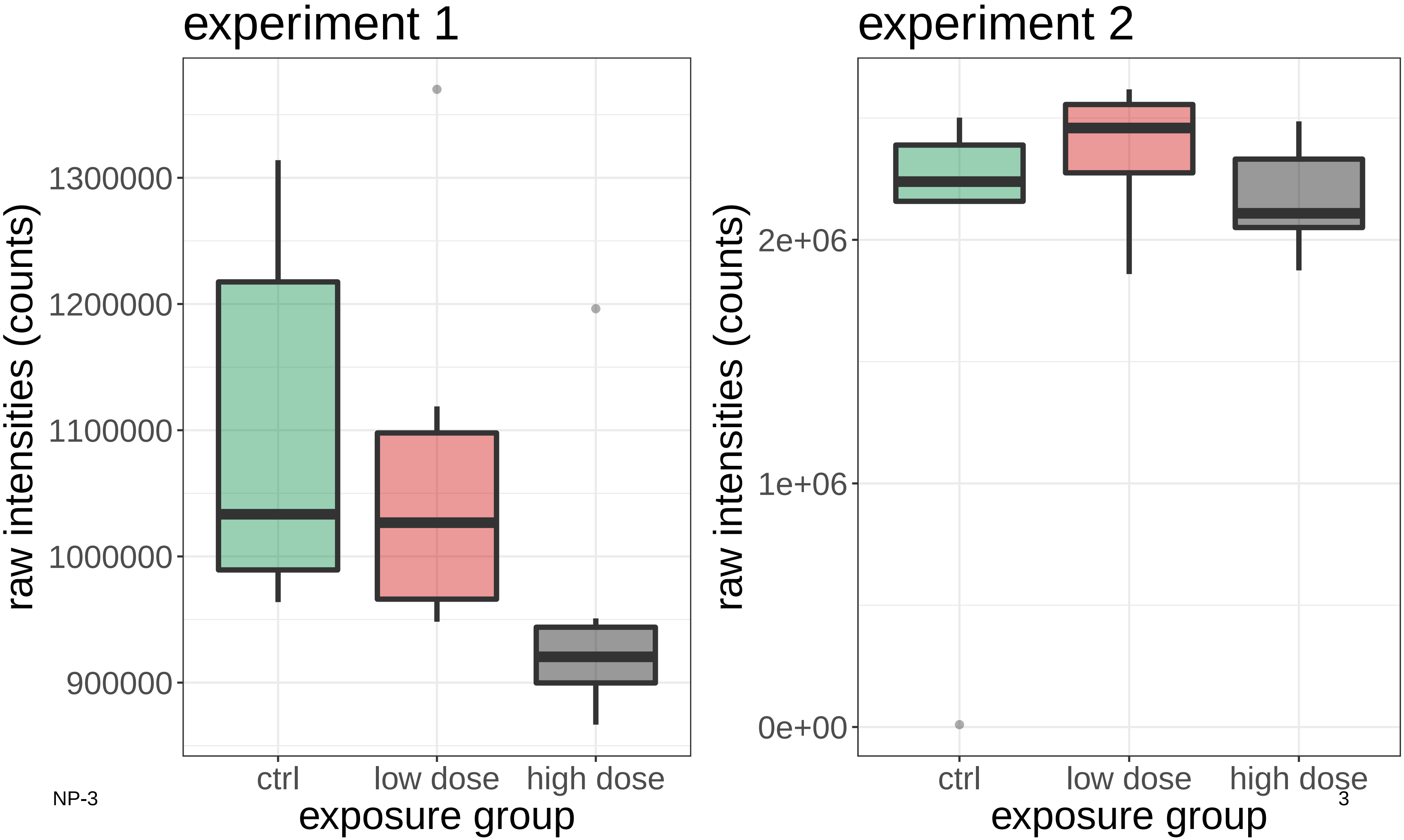
experiment 1
raw intensities (counts)
1300000
1200000
1100000
1000000
900000
ctrl
low dose
high dose
exposure group
experiment 2
raw intensities (counts)
2e+06
1e+06
0e+00
ctrl
low dose
high dose
exposure group

NP-3

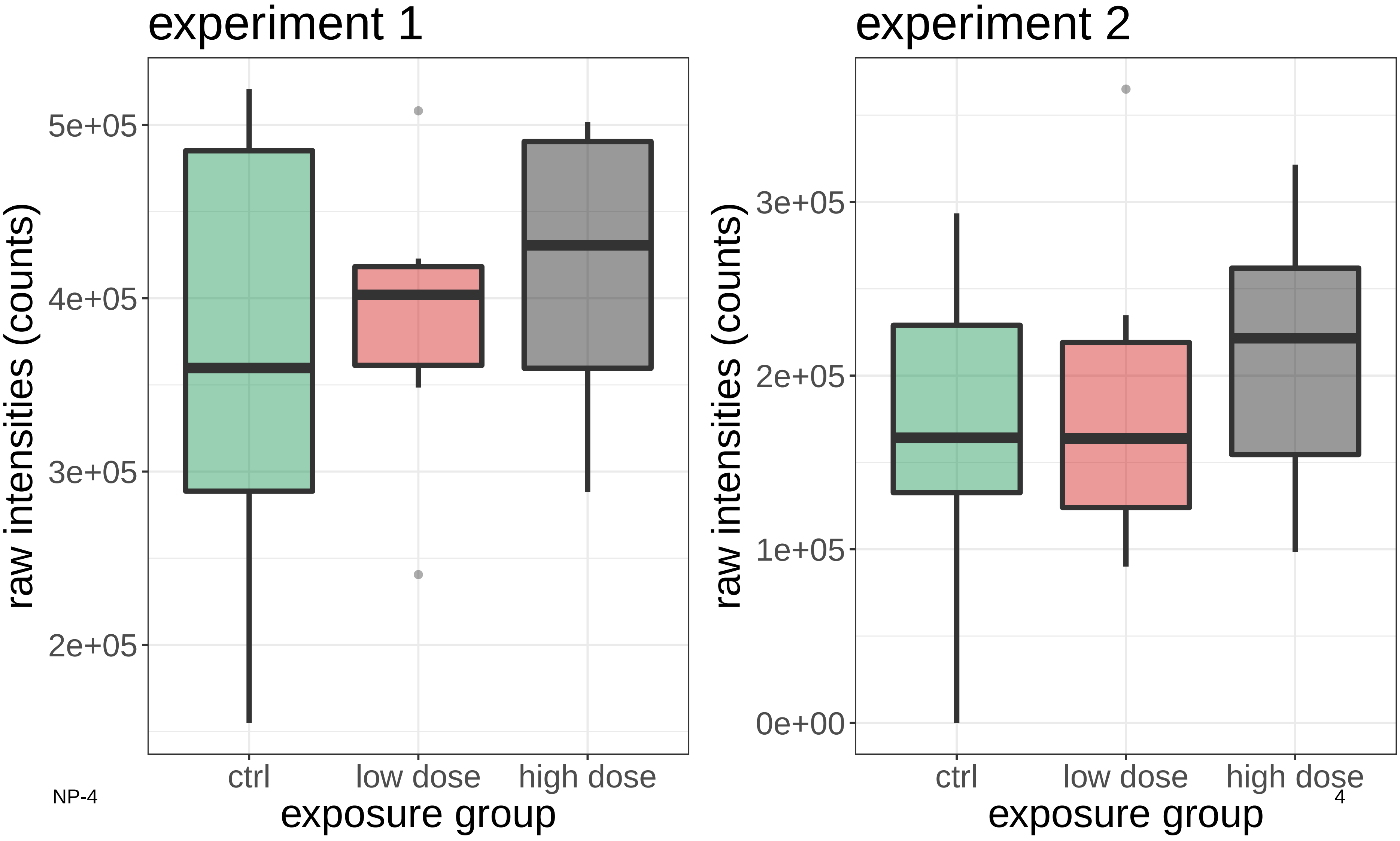
experiment 1
experiment 2
raw intensities (counts)
5e+05
4e+05
3e+05
2e+05
1e+05
0e+00
ctrl
low dose
high dose
exposure group

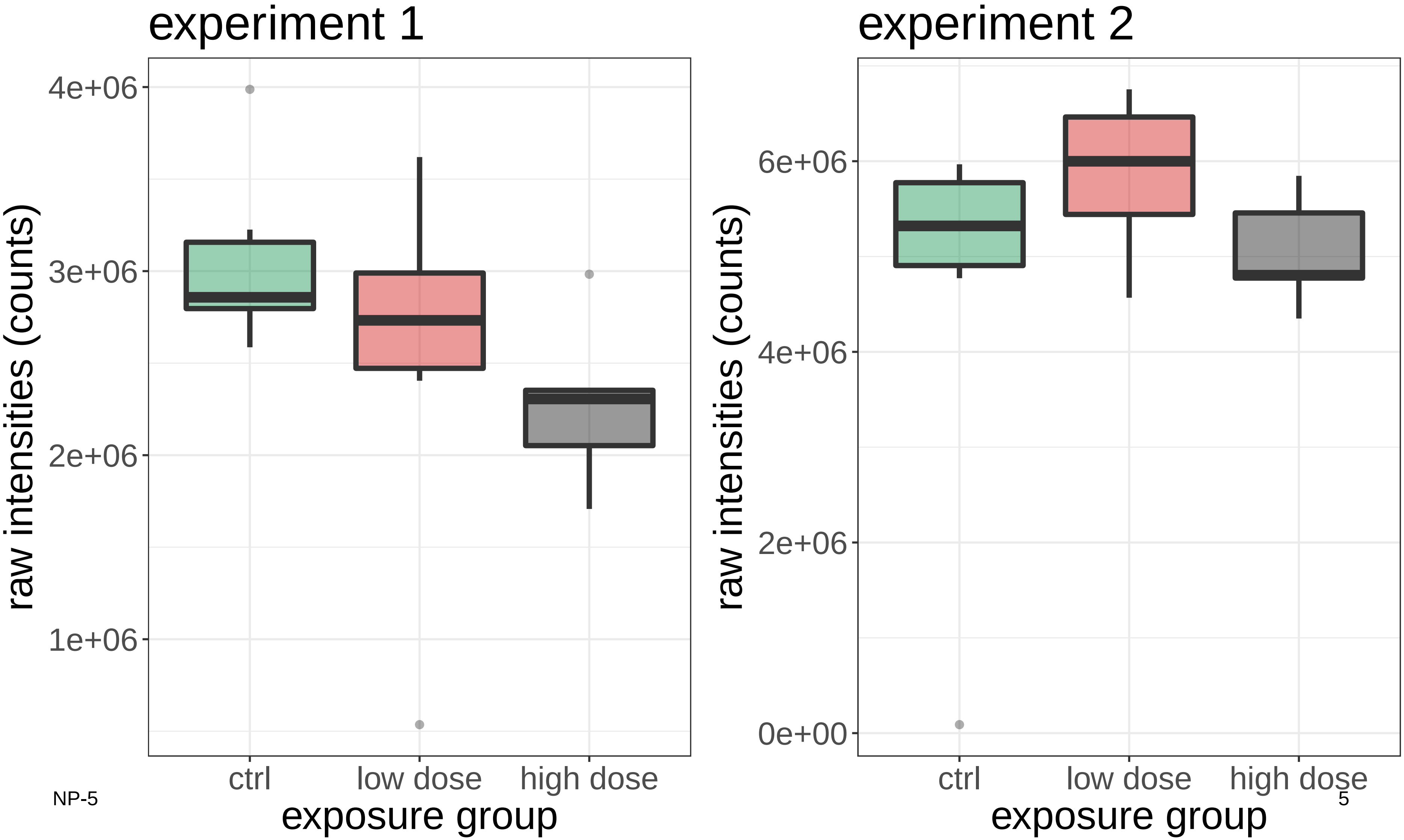
experiment 1
experiment 2
raw intensities (counts)
4e+06
3e+06
2e+06
1e+06
6e+06
4e+06
2e+06
0e+00
ctrl
low dose
high dose
exposure group

NP-5

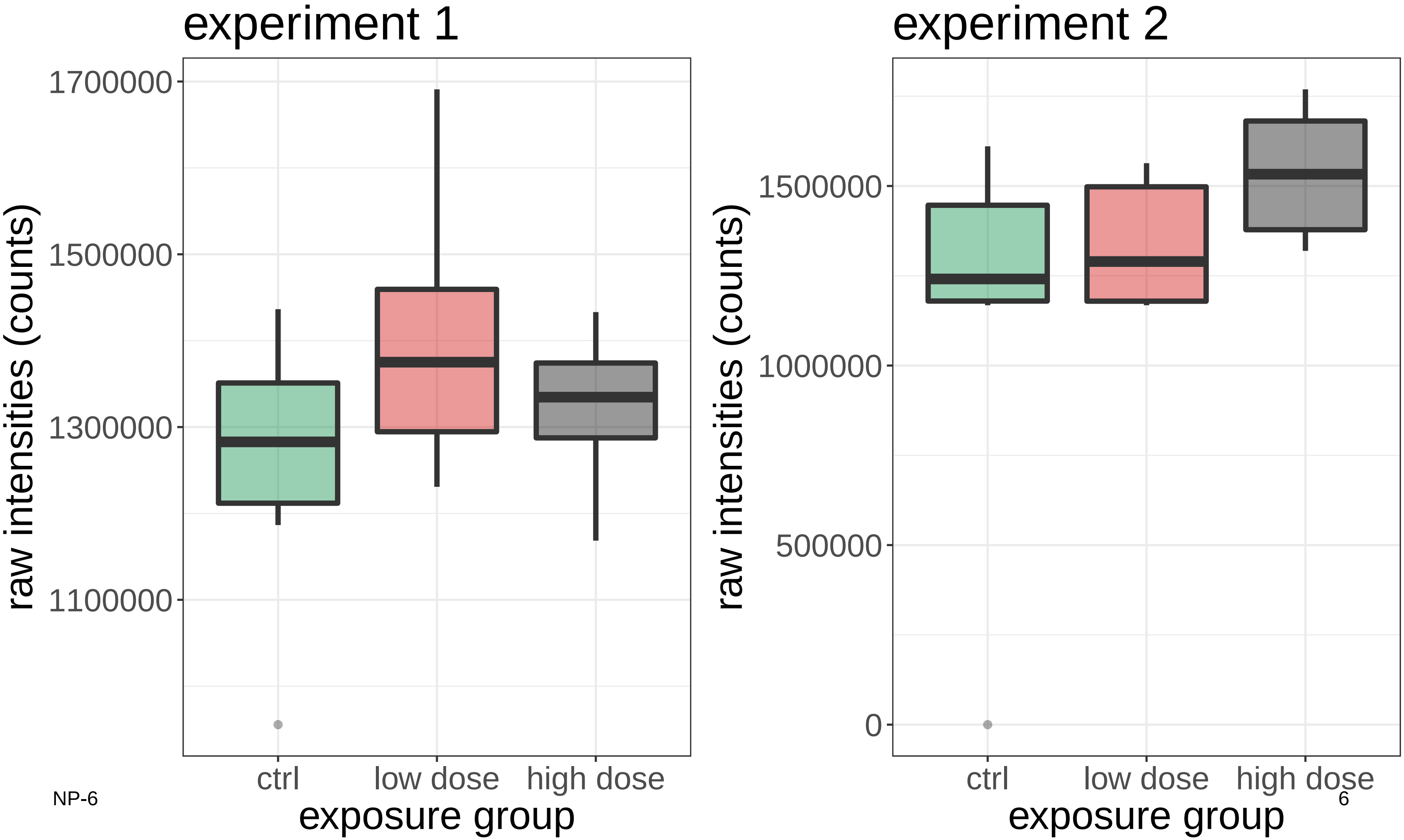
experiment 1
1700000
1500000
1300000
1100000
raw intensities (counts)
ctrl
low dose
high dose
exposure group
experiment 2
1500000
1000000
500000
0
raw intensities (counts)
ctrl
low dose
high dose
exposure group

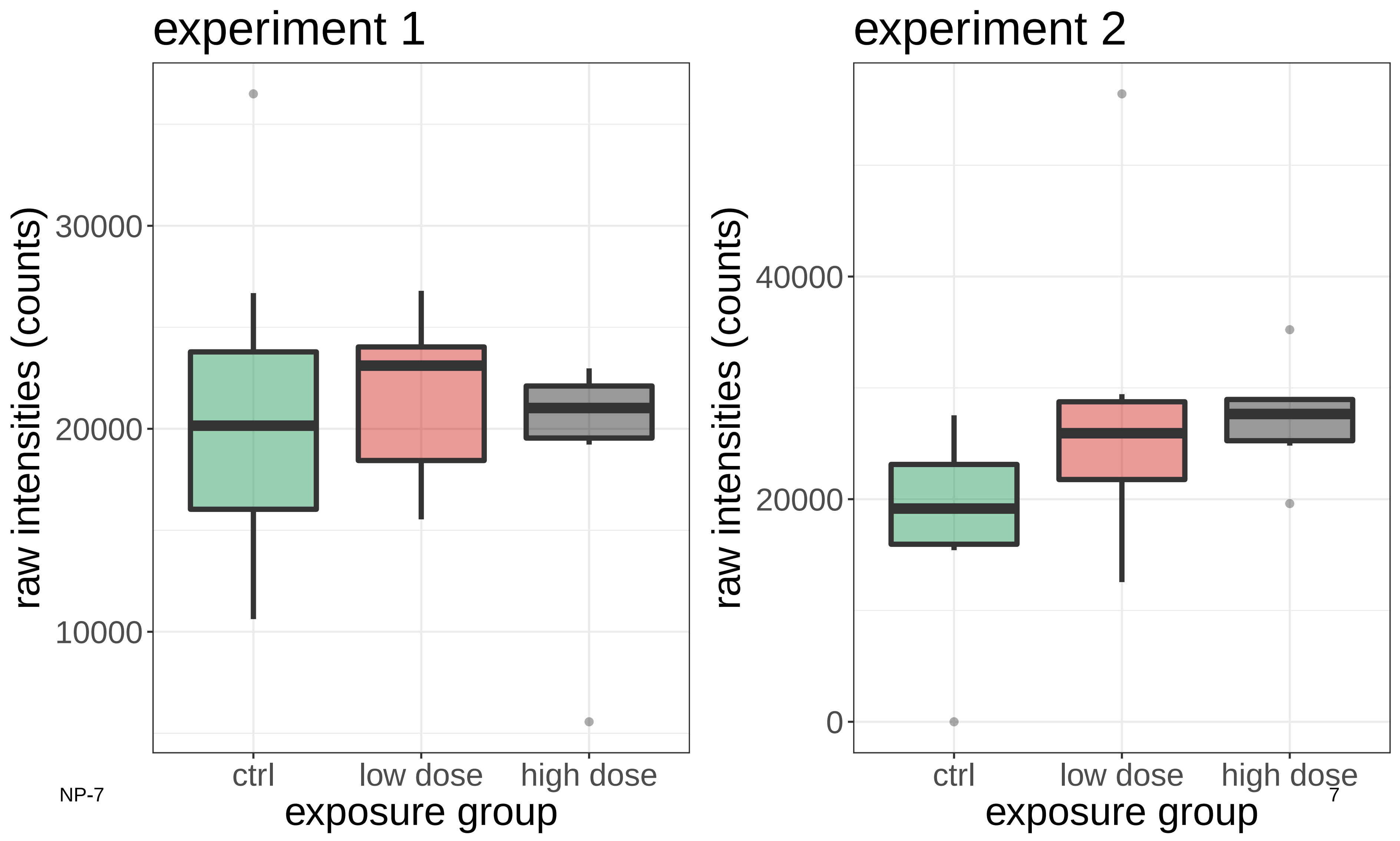
experiment 1
30000
20000
10000
raw intensities (counts)
ctrl
low dose
high dose
exposure group
experiment 2
40000
20000
0
raw intensities (counts)
ctrl
low dose
high dose
exposure group

NP-7

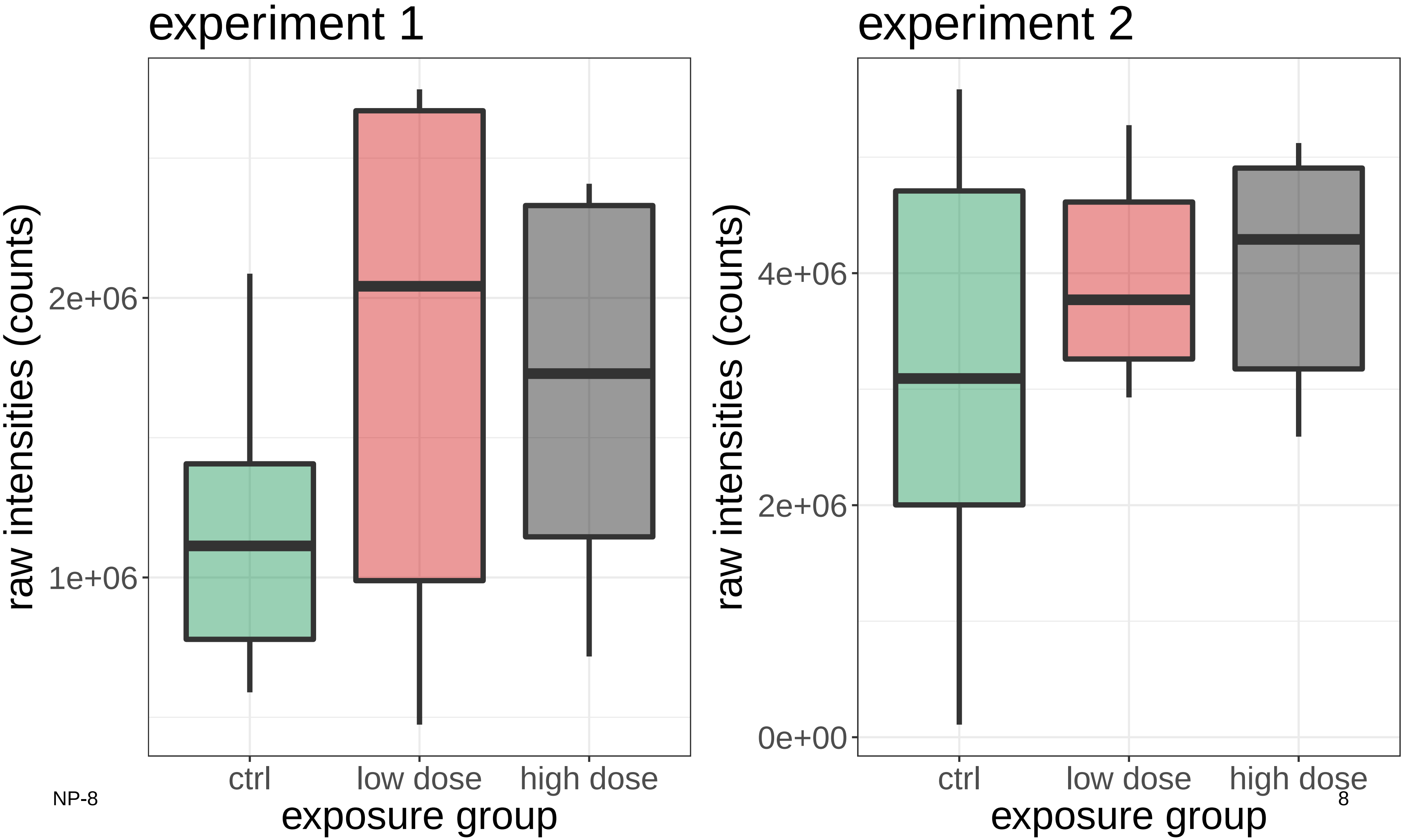
experiment 1
raw intensities (counts)
2e+06
1e+06
ctrl
low dose
high dose
exposure group
experiment 2
raw intensities (counts)
4e+06
2e+06
0e+00
ctrl
low dose
high dose
exposure group

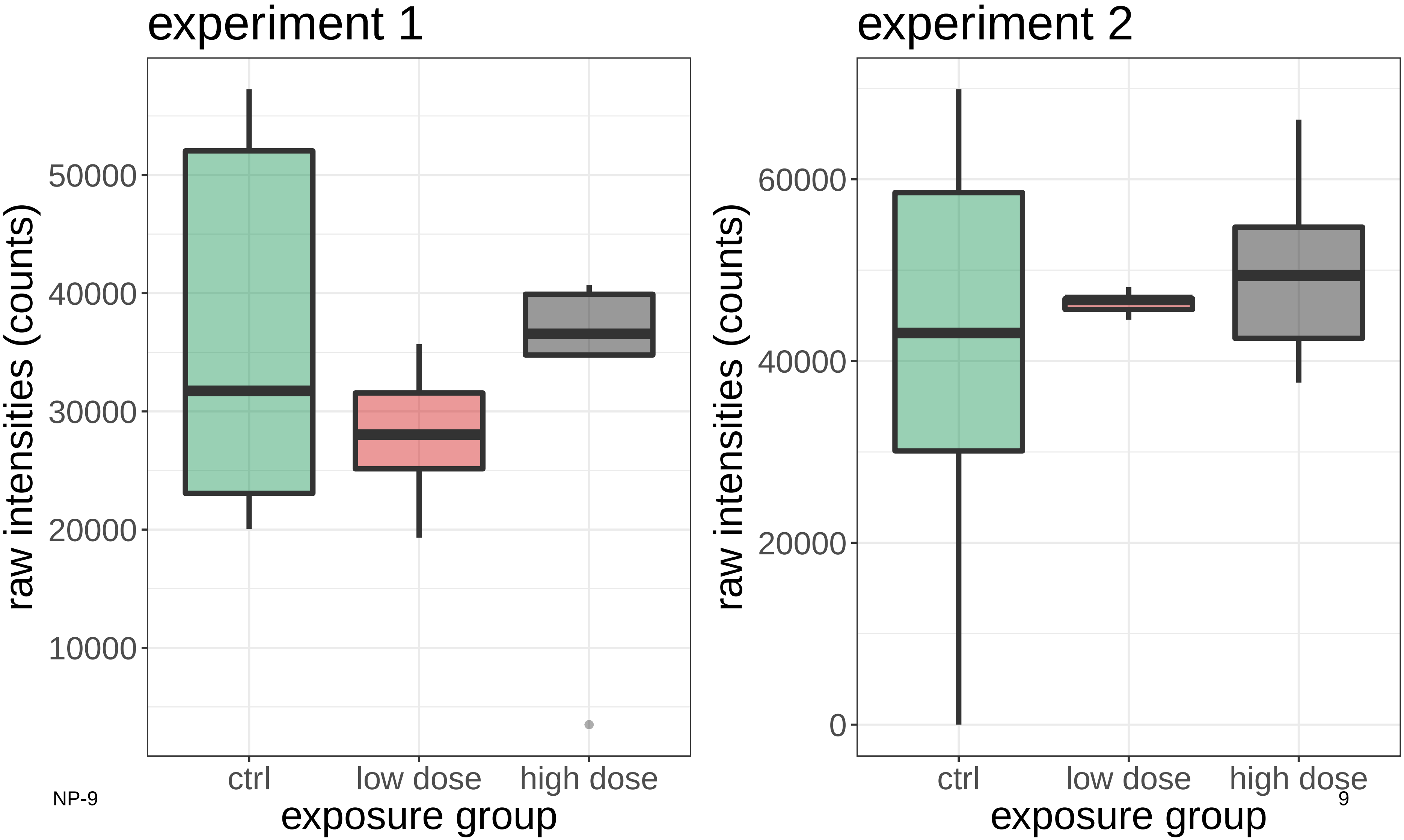
experiment 1
raw intensities (counts)
50000
40000
30000
20000
10000
ctrl
low dose
high dose
exposure group
experiment 2
raw intensities (counts)
60000
40000
20000
0
ctrl
low dose
high dose
exposure group

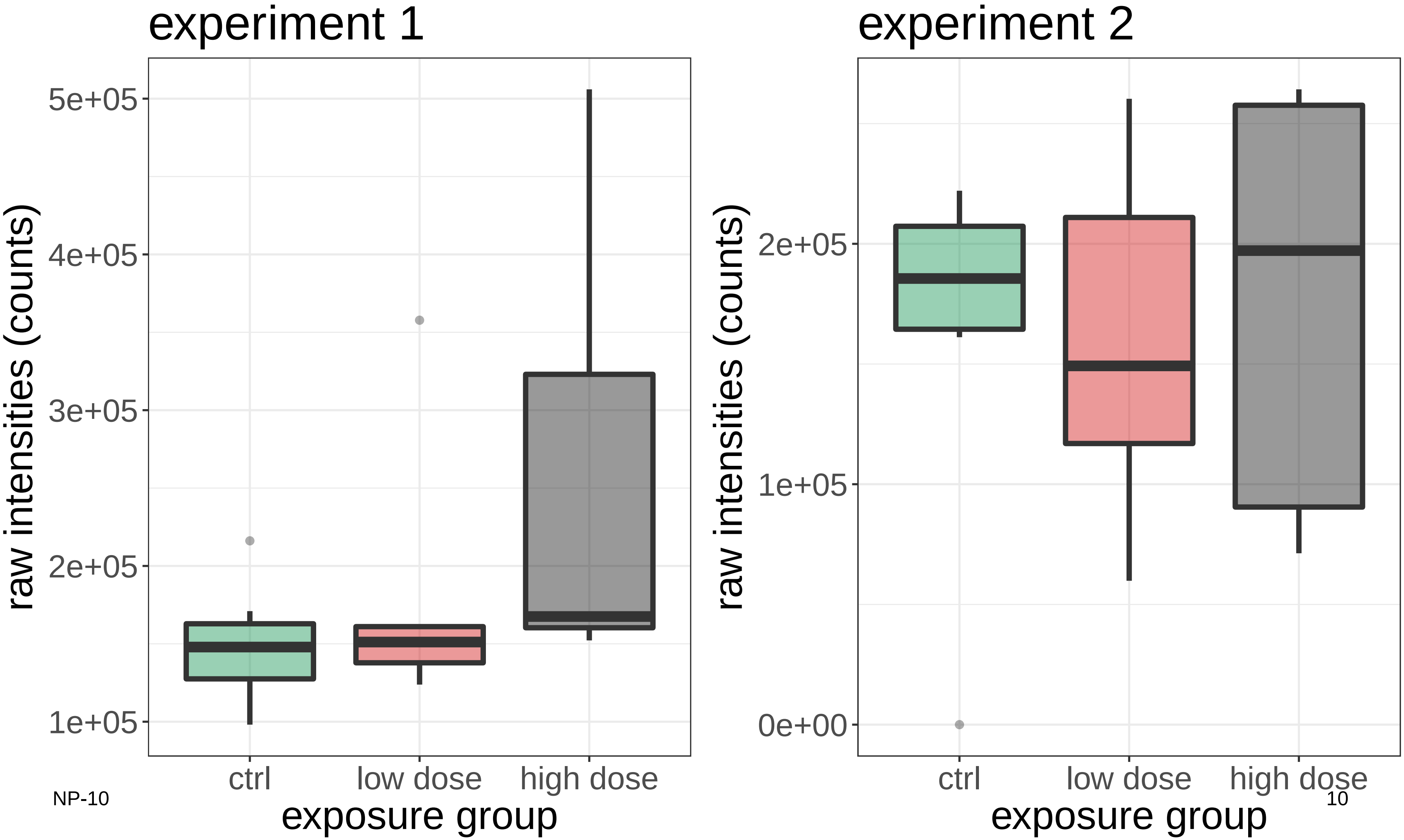
experiment 1
raw intensities (counts)
5e+05
4e+05
3e+05
2e+05
1e+05
ctrl
low dose
high dose
exposure group
experiment 2
raw intensities (counts)
2e+05
1e+05
0e+00
ctrl
low dose
high dose
exposure group

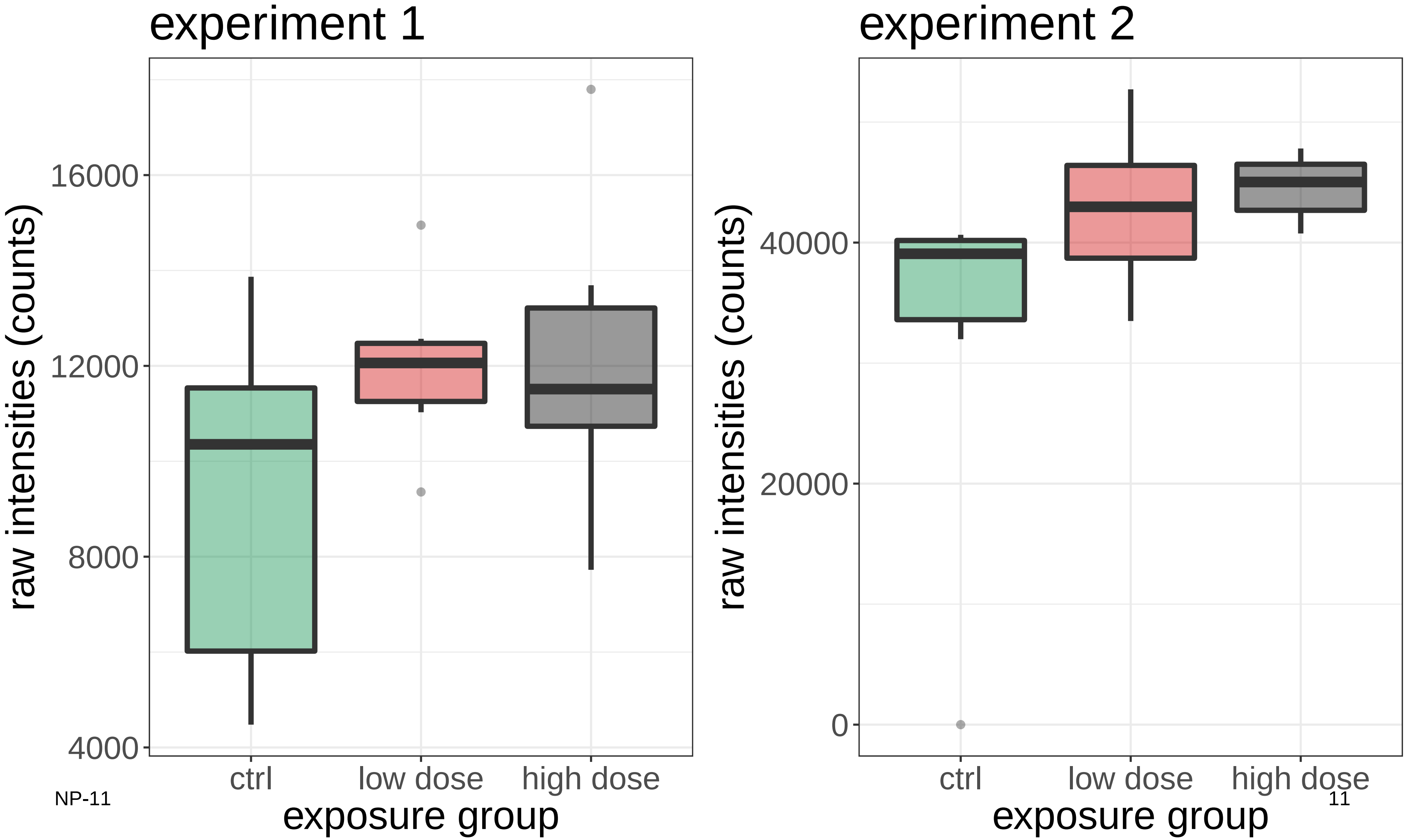
experiment 1
raw intensities (counts)
16000
12000
8000
4000
ctrl
low dose
high dose
exposure group
experiment 2
raw intensities (counts)
40000
20000
0
ctrl
low dose
high dose
exposure group

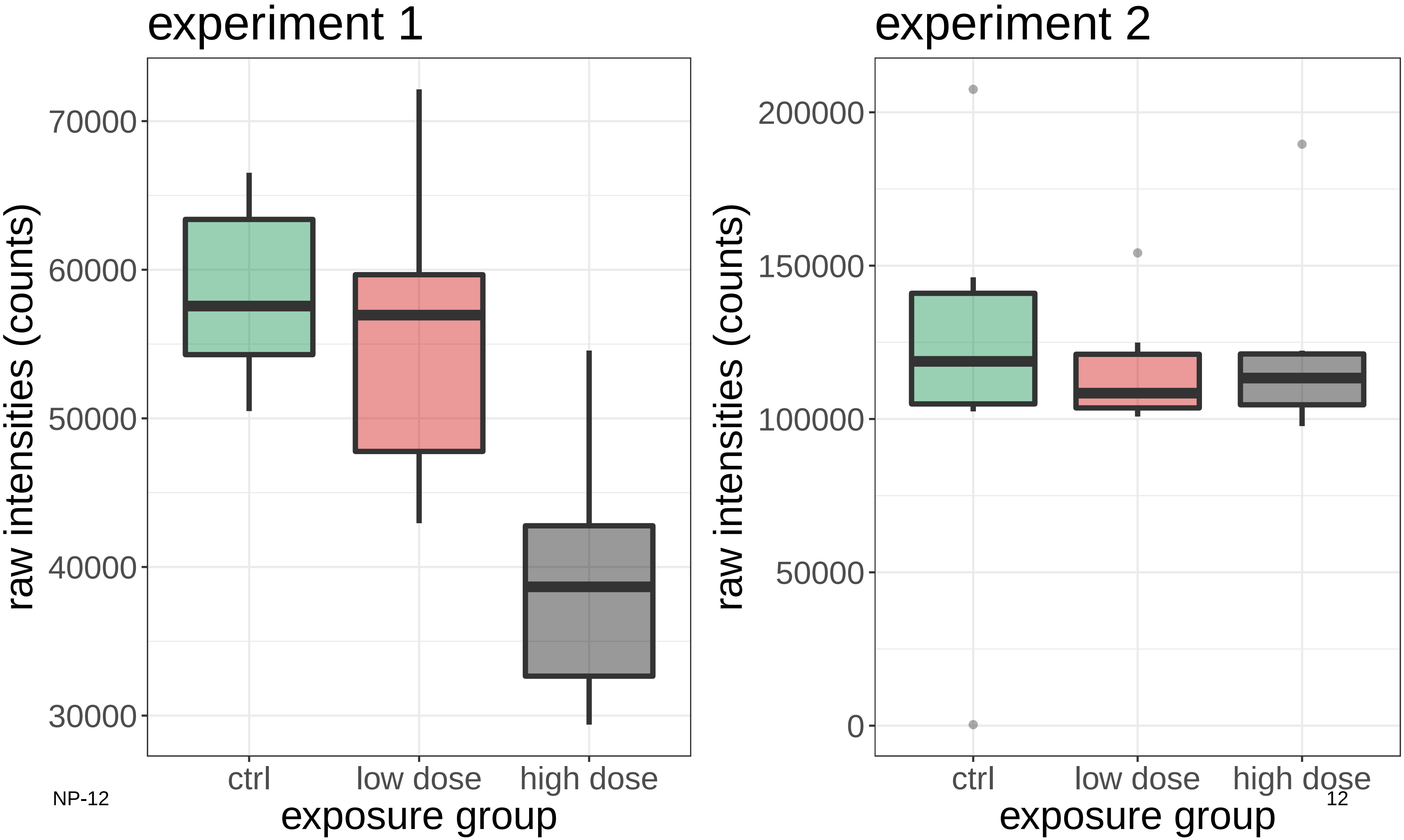
experiment 1
raw intensities (counts)
70000
60000
50000
40000
30000
ctrl
low dose
high dose
exposure group
experiment 2
raw intensities (counts)
200000
150000
100000
50000
0
ctrl
low dose
high dose
exposure group

NP-12

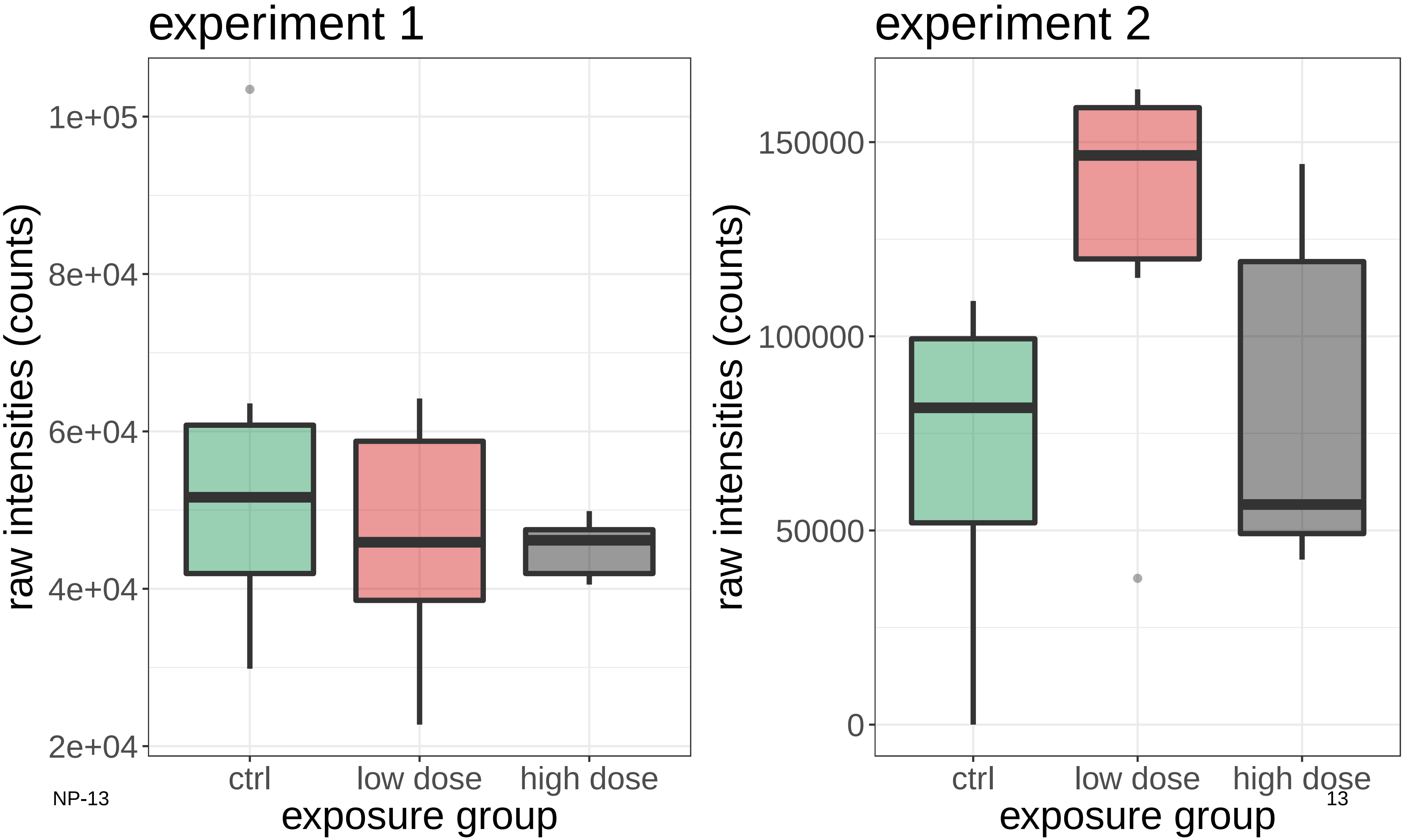
experiment 1
raw intensities (counts)
1e+05
8e+04
6e+04
4e+04
2e+04
ctrl
low dose
high dose
exposure group
experiment 2
raw intensities (counts)
150000
100000
50000
0
ctrl
low dose
high dose
exposure group

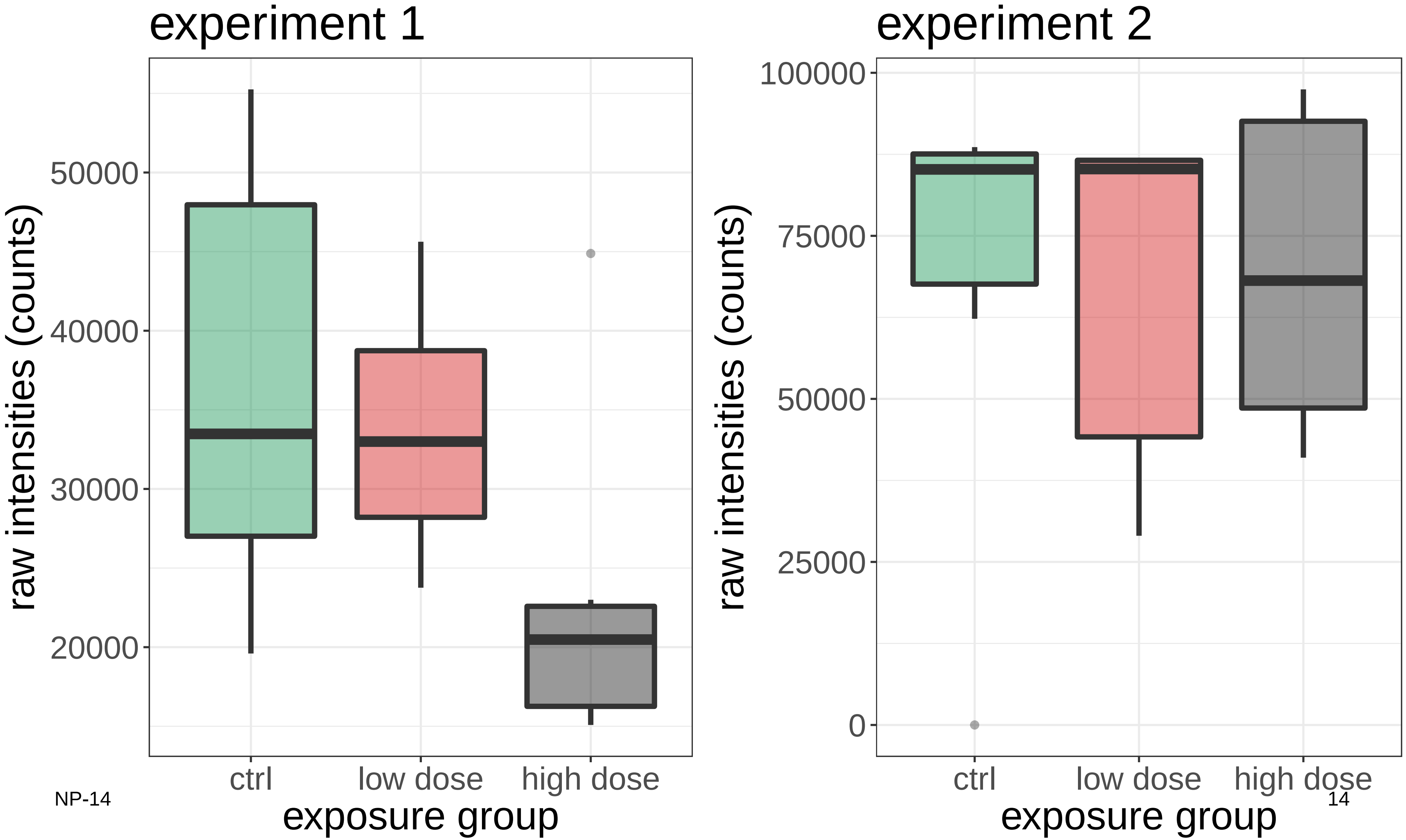
experiment 1
raw intensities (counts)
50000
40000
30000
20000
ctrl
low dose
high dose
exposure group
experiment 2
raw intensities (counts)
100000
75000
50000
25000
0
ctrl
low dose
high dose
exposure group

NP-14

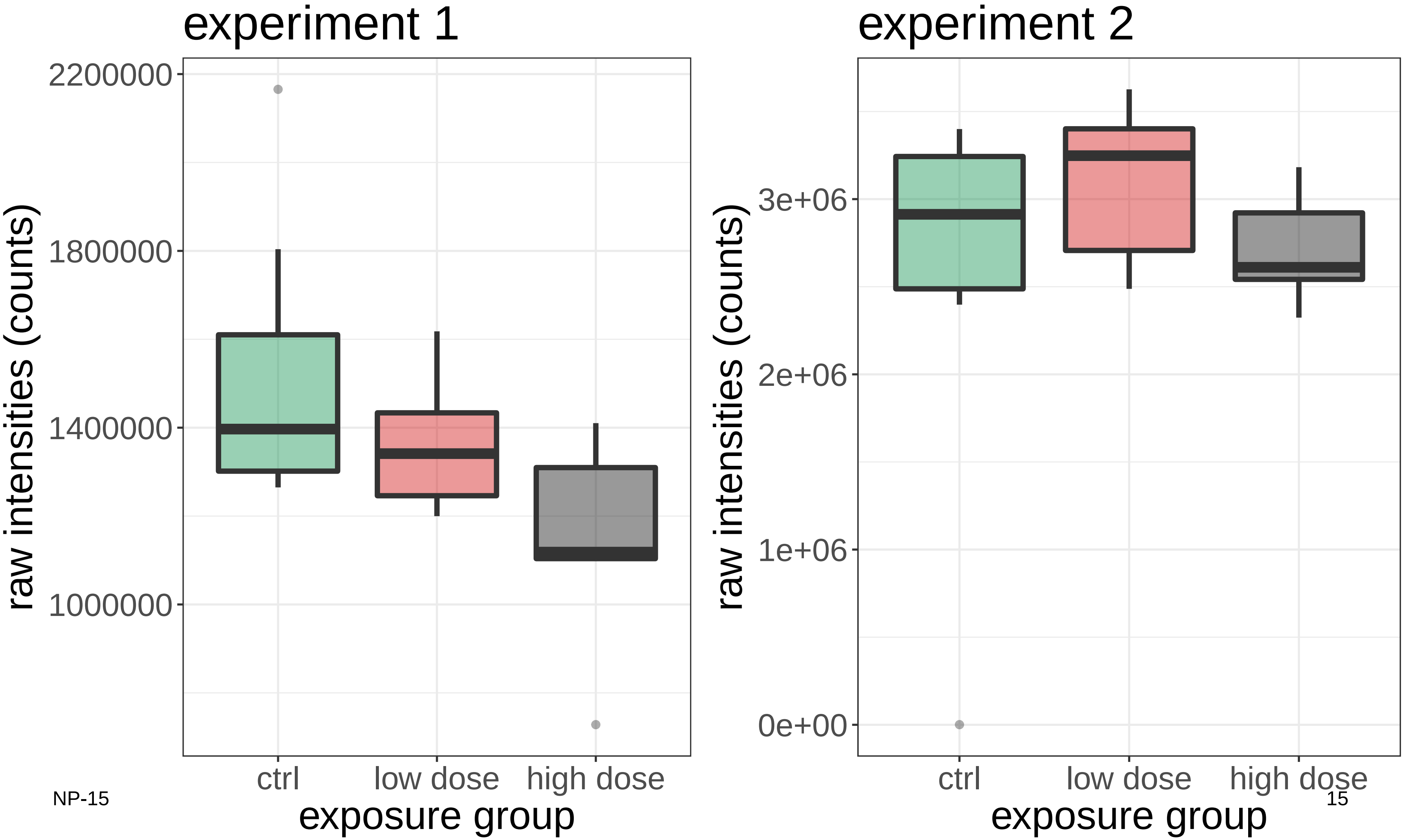
experiment 1
2200000
1800000
1400000
1000000
raw intensities (counts)
ctrl
low dose
high dose
exposure group
experiment 2
3e+06
2e+06
1e+06
0e+00
raw intensities (counts)
ctrl
low dose
high dose
exposure group

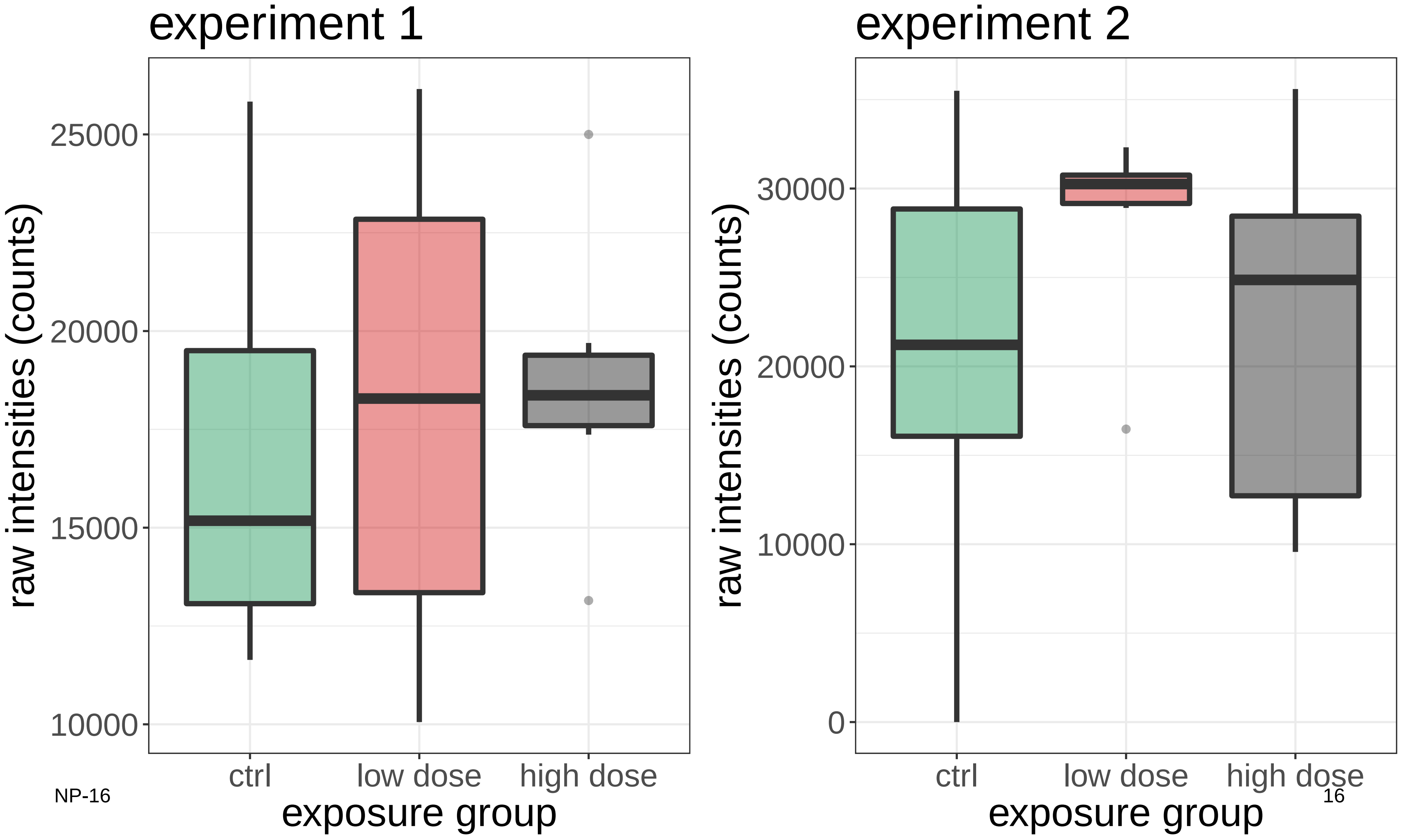
experiment 1
raw intensities (counts)
25000
20000
15000
10000
ctrl
low dose
high dose
exposure group
experiment 2
raw intensities (counts)
30000
20000
10000
0
ctrl
low dose
high dose
exposure group

NP-16

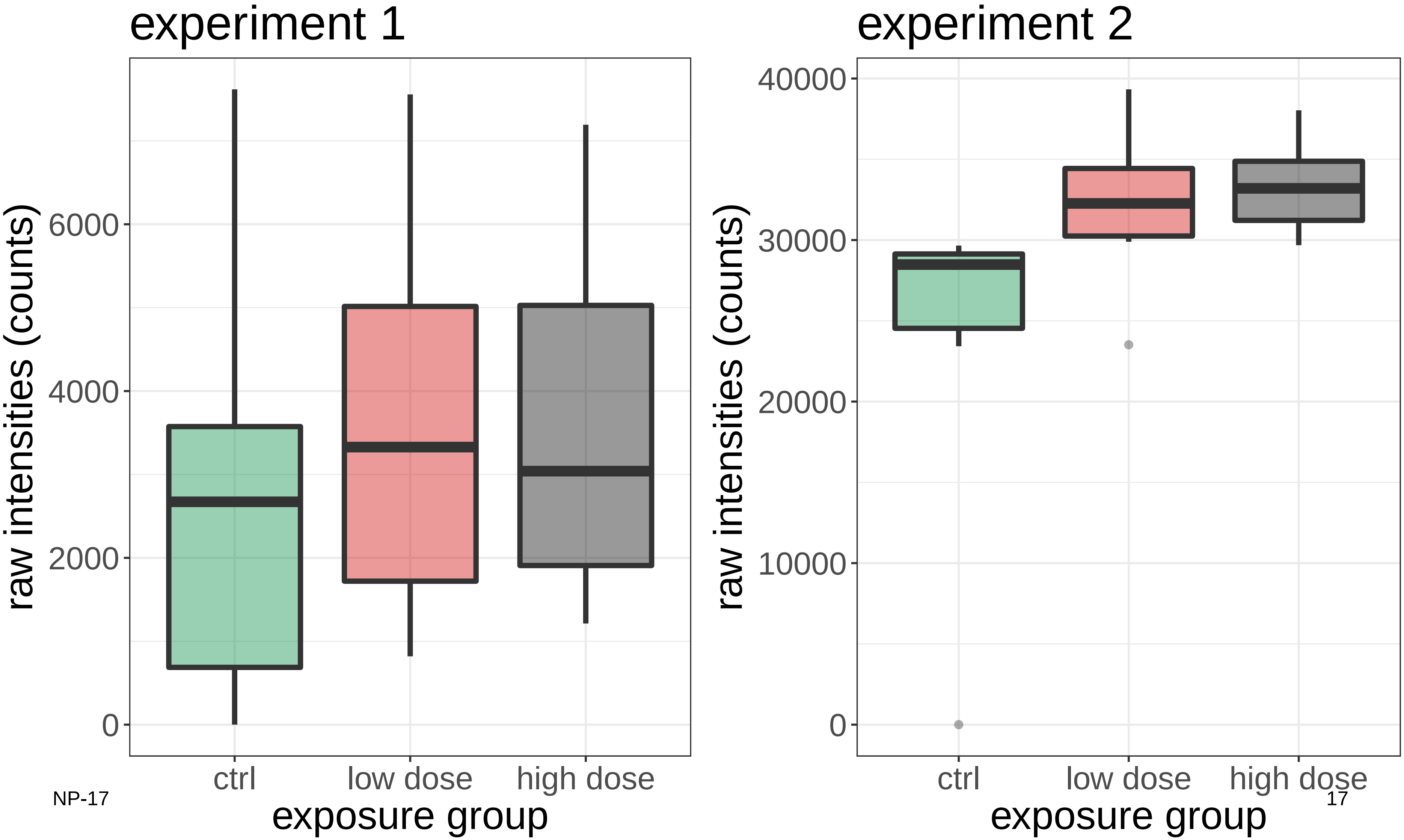
experiment 1
raw intensities (counts)
0
2000
4000
6000
ctrl
low dose
high dose
exposure group
experiment 2
raw intensities (counts)
0
10000
20000
30000
40000
ctrl
low dose
high dose
exposure group

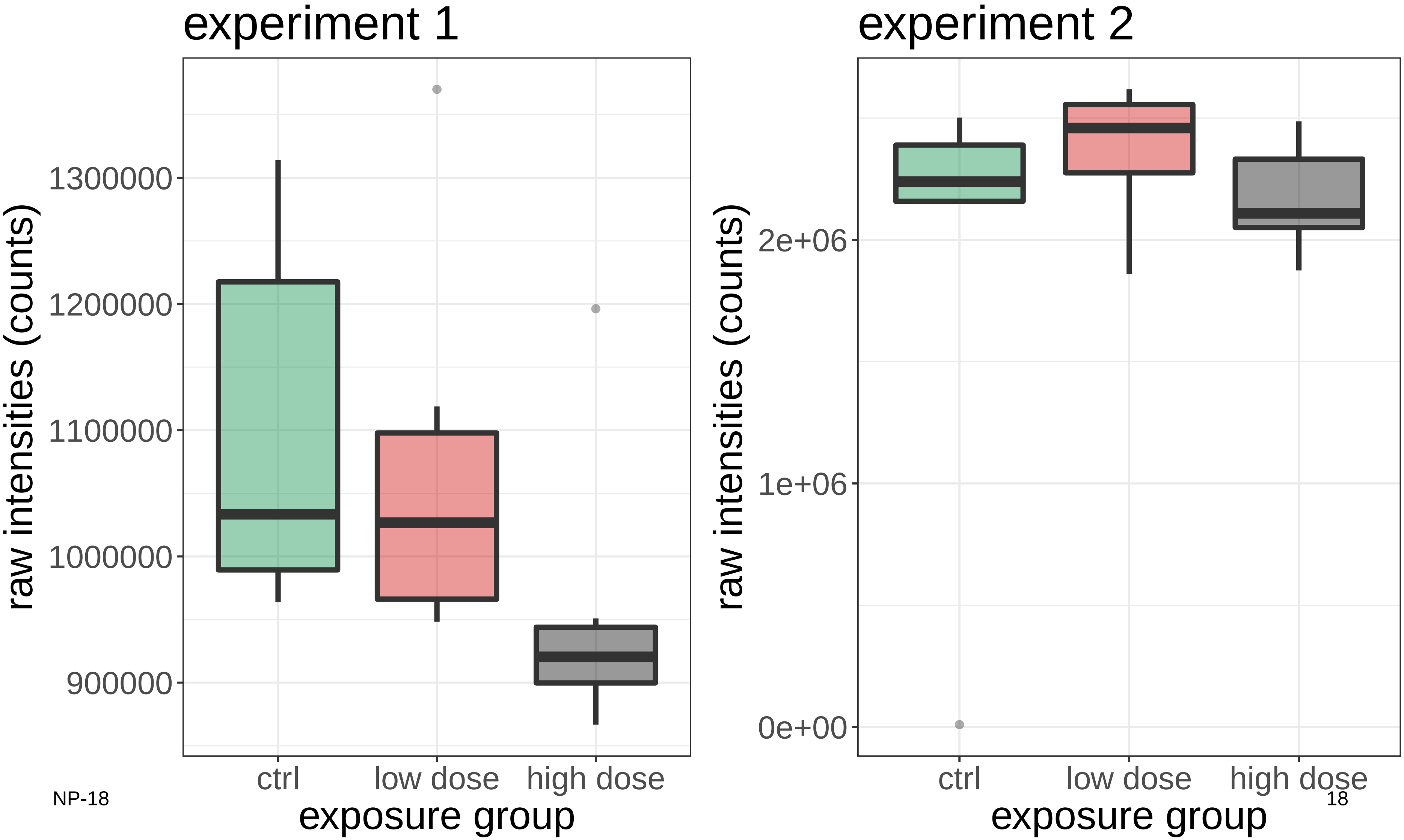
experiment 1
raw intensities (counts)
1300000
1200000
1100000
1000000
900000
ctrl
low dose
high dose
exposure group
experiment 2
raw intensities (counts)
2e+06
1e+06
0e+00
ctrl
low dose
high dose
exposure group
