# Supplementary material for: Exposure of HepaRG Cells to Sodium Saccharin Underpins the Importance of Including Non-Hepatotoxic Compounds When Investigating Toxicological Modes of Action Using Metabolomics
Source: Metabolites. 2019 Nov 4;9(11):265. doi: 10.3390/metabo9110265 (PMC6918164; doi:10.3390/metabo9110265)
Supplement: Supplementary file 1 [file metabolites-09-00265-s001.zip › supplementary materials/SI-R1.pdf]

Supplementary Information

Exposure of HepaRG cells to sodium saccharin indicates the importance of mechanistic interpretation during the investigation of toxicological modes of action

Matthias Cuykx[1,4]*, Charlie Beirnaert[2,3], Robim M Rodrigues[4], Kris Laukens[2,3], Tamara Vanhaecke[3,4$], Adrian Covaci[1$]*

[1] Toxicological Centre, University of Antwerp, Universiteitsplein 1, 2610 Wilrijk, Belgium

[2] Department of Mathematics & Computer Science, University of Antwerp, Middelheimlaan 1, 2020 Antwerp, Belgium

[3] Biomedical informatics network Antwerpen (biomina), University of Antwerp, Middelheimlaan 1, 2020 Antwerp, Belgium

[4] Research group *In Vitro* Toxicology and Dermato-Cosmetology (IVTD), Vrije Universiteit Brussel, Laarbeeklaan 103, 1090 Jette, Belgium

[$] shared Last author

* - corresponding authors: Matthias.Cuykx@uantwerpen.be; Adrian.Covaci@uantwerpen.be

fax: +32 3 265 27 22

Materials and Methods

SI-1: Standards and chemicals

Cryopreserved differentiated HepaRG® cells (HPRGC10) and recommended culture media and supplements (Basal Hepatic Cell Medium (MIL600), Thawing/Plating/General Purpose Medium Supplement with antibiotics (ADD670) and Additives for Maintenance/Metabolism with antibiotics (ADD620)) were obtained from Biopredic International (Rennes, France). Collagen type 1 was obtained from Corning (Wiesbaden, Germany). Dry ice was purchased from Strombeek IJsfabriek (Strombeek, Belgium). Ultrapure (milliQ) water was obtained by the use of a PURELAB device from Elga LabWater (Tienen, Belgium). Phosphate-buffered saline (PBS) was prepared on site at the research group *In Vitro* Toxicology and Dermato-Cosmetology (IVTD, VUB, Belgium).
The Lab-Tek Chamber Slide w/Cover Permanox Slide Sterile 2 well were purchased from Thermo Scientific (Rochester New York, VS). Sterile cell scrapers with a sharp edge (type GBO 541070) were purchased from Greiner Bio-One (Vilvoorde, Belgium). The equipment for the use of the TC10 Automated Cell Counter (Cell Counting Slides for TC10™/TC20™ Cell Counter, Dual-Chamber en Trypan Blue Dye, 0,40 % solution) were purchased from BioRAD Laboratories (Temse, Belgium).
Sodium saccharin was purchased at Certa nv (Braine-l'Alleud, Belgium).

Ammonium acetate (for analysis, >98 %) (NH_4Ac), formic acid (for analysis, >98 %) (FA), acetic acid (glacial, anhydric for analysis, 100 %) (HAc), chloroform (for analysis) ($CHCl_3$), ammonium carbonate (extra pure), 2-propanol (for analysis) (isopropanol, IPA) and neutral red (NR) were purchased from Merck KGaA (Darmstadt, Germany). Acetonitrile (HPLC grade) (ACN) and methanol (LC-MS grade) (MeOH) were obtained from Fisher (Loughborough, VK). Ammonium formate (97 %) (NH_4F), L-ascorbic acid (BioXtra, >99 %), butylhydroxytoluene (>99 %) (BHT) and ethylenediaminetetraacetic acid (trace metal basis, 99,995 %) (EDTA) were purchased from Sigma-Aldrich (Steinheim, Germany).
The isotope labeled standards D-tryptophane-2',4',5',6',7'-d5 (98 %), laurylic acid-12,12,12-d3 (99 %) and cholesterol-25,26,26,26-d4 (99 %) were obtained from CDN Isotopes (Pointe-Claire, Quebec, Canada). The standards 1,2-diheptadecanoyl-sn-glycero-3-phosphocholine (PC-17:0), 1,2-diheptadecanoyl-sn-glycero-3-fosfaat (PA-17:0), N-heptadecanoyl-sphing-4-enine (Cer-17:0) and 1-heptadecanoyl-2-hydroxy-sn-glycero-3-phosphocholine (LPC-17:0) were purchased from Avanti Lipids (Alabaster, Alabama, VS). ATP-$^{13}C_{10}$, TG-($^{12}C_{15}$-^{13}C:0)$_3$, lysine-$^{13}C_6$-$^{15}N_2$, glucose-$^{13}C_6$, ADP, ATP, stearic acid, folic acid, mono-, di- and trioleylglycerol, misoprostol, cholic acid-d4, phosphoenolpyruvate, ornithine, glutamate-d4, leucine-d3, adenine, glucosephosphate, citric acid, caffeine, N-acetylglucosamine, pyridoxal-d3, dopamine-d4, palmitoylcarnitine, cholesterylpalmitate, succinic acid-d4 and a standardised amino acid mix (AAS18) were purchased from Sigma-Aldrich (St. Louis, Missouri, VS).

SI-2: Determination of testing concentrations

The IC_{10} dosage for a 24 h and 72 h exposure were assessed with a Neutral Red Uptake assay (NRU).[1,2]

The differentiated HepaRG® cells were seeded in collagen-coated 96 well plates at a density of 89 x 10^3 cells/well using a media consisting of Basal Hepatic Medium and the HepaRG® thawing, seeding and general use supplement (ADD670). The incubation medium was replaced by a Maintenance medium which consisted of Basal Hepatic Medium and the Maintenance and metabolism supplement (ADD620) 24 h after seeding (day 2). The maintenance medium was renewed again at day 4 and day 7. After seven days of cultivation, the wells were divided in 2 control groups and 8 groups which are exposed to 100 µL of Medium containing sodium saccharin at different concentrations ranging from 62.5 µg/mL to 8,000 µg/mL (Table SI-2.1) for a period of 72 h. The medium was replaced every 24 h during the 72 h exposure to simulate a repeated dose exposure. The cells were incubated at 37 °C, 5 % CO_2 and saturating humidity.

Table SI-2.1: Concentration range (in µg/mL) for the NRU-assay of sodium saccharin on HepaRG cells for a period of 72 h.

	C1	C2	C3	C4	C5	C6	C7	C8
72 h	8,000	4,000	2,000	1,000	500	250	125	62.5

After exposure, the cells were washed with PBS and incubated at 37 °C and saturating humidity with 250 µL medium containing 25 µg/mL Neutral Red. The medium was removed after three hours of incubation. The precipitated neutral red was resolubilised for one hour in 100 µL of a 1 % (*v/v*) HAc solution in 1/1 ethanol and water (*v/v*) and the absorption was measured at 540 nm using a Perkin Elmer Precisely 1420 Victor³ Multilabel Counter (Perkin Elmer, Zaventem, Belgium). The absorbance values were transferred to Masterplex® 2010 (versie 2.0.0.73, Mirai Bio Group of Hitachi Solutions America) to plot a dose-response curve. The response curves of the three plates shown in Figure SI-2.1 indicate cell death occurring from 2,000 µg/mL. Since sodium saccharin is considered not to be hepatotoxic, the observed cell death is probably related to high osmotic pressure generated by the high dosage of the molecule.

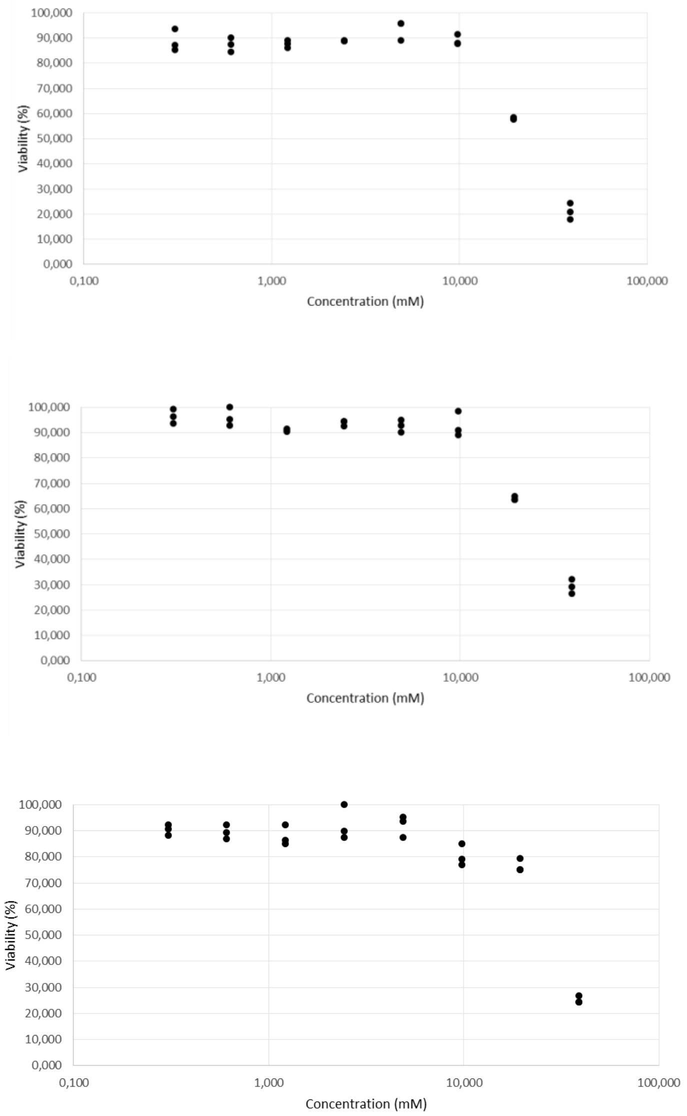

Figure SI-2.1: Viability curves for the NRU-assay 72 h of exposure. Cytotoxicity is observed from 2,000 µg/mL, which is equal to an osmotic pressure of ± 20 mOsm/L.

SI-3: Protocols

Cell cultivation and exposure

All solutions and materials used were sterilized and all procedures were conducted aseptically in an MSC 1.2 Advantage LAF cabinet (Thermo Scientific, Langenselbold, Germany). Cryopreserved differentiated HepaRG® cells were thawed and seeded in collagen-coated 2-well Lab-Tek chamber slides (Permanox, Sigma Aldrich) at a density of 1.03 x 10^6 cells/well using a media consisting of Basal Hepatic Medium and the HepaRG® thawing, seeding and general use supplement (ADD670). For biological analysis 18 wells were seeded and no cells were added to 2 additional blank chamber slides. The cultures were placed in a Galaxy® 170 S incubator (Eppendorf, Hamburg, Germany) at 37 °C, 5 % CO_2 and a saturated humidity. The incubation medium was replaced by a maintenance medium which consisted of Basal Hepatic Medium and the Maintenance and Metabolism supplement (ADD620) 24 h after seeding (day 2). The maintenance medium was renewed again at day 4 and day 7.

After seven days of cultivation, the cell cultures were visually checked for hepatocyte/biliary cell ratio and block randomised in 3 groups of 6 replicates: a negative control group in comparison to a dose of sodium saccharin at the high-dose concentration of 1000 µg/mL and a 1/10 dilution of the high-dose concentration i.e. 100 µg/mL. The cultures were exposed for 72 h in a repeated dose exposure.

Sample preparation

The cell cultures were harvested and processed by the protocol of Wu et al. and adapted by Cuykx and Mortelé et al.[3,4] After exposure, the cells were visually checked using a NIKON microscope with a 10x10 and 10x20 magnification. The cells were washed twice with PBS (37 °C) and flash frozen on liquid nitrogen. The cells were scraped from the surface with three times 200 µL of a cooled (-80 °C) 80 % (*v/v*) MeOH/milliQ water solution, quenching metabolism and precipitating proteins. The cell extracts were collected in a precooled vial (-20 °C) which contained 420 µL chloroform and 500 µL ultrapure water, stabilised with antioxidant (1 mM of BHT and vitamin C) and chelating additives (0.5 mM $(NH_4)_2EDTA$ (final solvent ratio 2/3/2 water/MeOH/$CHCl_3$). 40 µl of internal standards (1 ppm final concentration) was added to the mixture and the samples were vortexed until an emulsion was formed and vortexed another two times for 30 s. After vortexing, the vials were equilibrated for 10 minutes and centrifuged at 2200 g for 7 min with slow deceleration by an Eppendorf Centrifuge 5804 (Eppendorf, Hamburg, Germany).

Two times 400 µL of the polar and two times 100 µL of the non-polar fraction were recovered. A QC was created by collecting 40 µL and 20 µL aliquots of all samples for the polar and non-polar phases respectively. The polar fraction was dried under vacuum using a Savant Speedvac concentrator SVC 100 H (Savant, Thermo Scientific, USA), the apolar fraction was evaporated under a nitrogen stream. All dry samples were stored at -80 °C.

SI-4: parameters of the LC-QTOFMS acquisition platforms

The samples of the apolar phase were resuspended in 50 µL of a 35/65 (v/v) IPA/MeOH solution, the polar samples in 40 µL of a 60/40 (v/v) ACN/water solution. The apolar QC-pools were resuspended in 190 µL 35/65 (v/v) IPA/MeOH, the polar QC-pools were resuspended in 90 µL 60/40 (v/v) of ACN/water. The samples and QC-pools were filtered over a 0.22 µm centrifugal filter by use of the Microfuge 18 centrifuge (Beckman Coulter, Suarlée, Belgium) for 2 min at 14 000 g. 20 µL of the samples were transferred in a Greiner 384 well plate for LC-MS analysis, the QC-pools were entirely transferred to LC-vials with inserts. The well plates and LC-vials with inserts were sealed and placed in the LC autosampler at 4°C.

All measurements were conducted using the Agilent 1290 infinity UHPLC and the Agilent 6530 Q-TOF-MS with Agilent jet-stream-electrospray ionisation (AJS-ESI) (Agilent Technologies, Santa Clara, Californië, VS).

For the analysis of the non-polar phase in positive and negative ionisation mode, the separation was conducted using a Kinetex® XB-C18 column (150 x 2.1 mm, 1.7 µm particle size, Phenomenex, Utrecht, The Netherlands). For analysis of the negative ionised apolar phase, mobile phase A consisted of 50/50 (v/v) MeOH / 10 mM NH_4Ac in milliQ water (pH 6.7 ± 0.05). Mobile phase B consisted of 2/10/88 (v/v) 10 mM NH_4Ac in milliQ water / MeOH / IPA. Metabolites were separated by a gradient elution starting at 55 % B for 1 min, followed by a linear gradient to 70 % B at 5 min. Afterwards, another gradient increased the percentage of B to 91 % at 20 min. The column was flushed with 100 % for 5 min and re-equilibrated at starting conditions for 9 min. The flow rate was 0.25 mL/min and the column was heated to 55 °C to reduce backpressure. The QTOF was set an acquisition range of 100 – 1400 m/z, the source conditions were as follows: drying and sheath gas were set at a temperature of 325 °C and a flow of 8 L/min. The nebuliser pressure was 30 psig, nozzle voltage was set at 0 V. The capillary voltage was set at 3750V. The acquisition was performed in MS-only mode at a scan rate of 2.5 spectra/s for the samples. MS/MS spectra were acquired during the equilibration runs using auto-MS/MS (data-dependent acquisition) at a scan rate of 6.67 spectra/s using a threshold of minimal 10 000 counts and a maximum of 12 precursors per scan cycle. The quadrupole was set at a small width (1.3 amu), the collision cell was set at 10, 20 and 40 V.

For the analysis of the positive ionised non-polar phase, mobile phase A consisted of 50/50 (v/v) ACN / 5 mM NH_4Ac and 0,1 % HAc in milliQ water (pH 4,2 ± 0,05). Mobile phase B consisted of 2/10/88 (v/v) 5 mM NH_4Ac and 0,1 % HAc in milliQ water/ACN/IPA. The analytical run started at 55 % of B for 1 min, followed by a 4-min gradient to 70 % B. Afterwards, a new gradient increased the percentage of B to

98 % at 25 min, followed by an 8 min rinse. Afterwards, the column was re-equilibrated at starting conditions for 5 min. The flow rate was 0.25 mL/min at a temperature of 55 °C. The QTOF was set an acquisition range of 100 – 1400 m/z, the source conditions were as followed: drying and sheath gas were set at a temperature of 325 °C and a flow of 8 L/min. The nebuliser pressure was 30 psig, nozzle voltage was set at 500 V. Capillary voltage was set at 3500 V. Acquisition was performed in MS-only mode at a scan rate of 4 spectra/s for the samples. MS/MS spectra were acquired during the equilibration runs using auto-MS/MS (data-dependent acquisition) at a scan rate of 6.67 spectra/s using a threshold of minimal 10 000 counts and a maximum of 12 precursors per scan cycle. The quadrupole was set at a small width (1.3 amu), the collision cell was set at 10, 20 and 40 V.

For the polar samples, the analytes from the negative ionisation modus were separated using an iHILIC FUSION polymer column (100 x 2.1 mm, 5 µm particle size, HILICON AB, Umeå, Sweden). Mobile phase A consisted of a 10 mM $(NH_4)_2CO_3$ solution in milliQ (pH 9.00 ± 0.05), mobile phase B contained 20/80 (v/v) MeOH/ACN. The chromatographic analysis started at 95 % B for 3 min, followed by a linear gradient to 60 % B at 10 min. The column was rinsed with 20 % B for 8 min and re-equilibrated at starting conditions for 15 min. The flow rate was 0.15 mL/min and column temperature was 30 °C. The QTOF scanned at a range of 65 to 1100 m/z in MS only at a scan rate of 2 spectra/s. Drying gas temperature and flow were 250 °C at 10 L/min and sheath gas parameters were set at 350 °C at 10 L/min. The nebuliser had a pressure of 45 psig, capillary voltage was set at 2 000 V and no nozzle voltage was applied. MS/MS spectra were acquired during the equilibration runs using auto-MS/MS (data-dependent acquisition) at a scan rate of 8 spectra/s using a threshold of minimal 1 000 counts and a maximum of 12 precursors per scan cycle. The quadrupole was set at a small width (1.3 amu) and the collision cell was set at 10, 20 and 40 V.

In positive ionisation mode, the analytes were separated using an iHILIC Fusion mixed mode column (100 x 2.1 mm, 1,8 µm particle size, HILICON AB, Umeå, Sweden). Mobile phase A contained 10 mM NH_4F and 0.1 % (v/v) FA in milliQ water (pH 3.15 ± 0.05), mobile phase B contained 2/98 (v/v) MeOH/ACN. Chromatographic separation started at 95 % B for 2 min, followed by a gradient to 65 % B at 8 min and 25 % B at 13 min. The column was rinsed at 25 % B for 5 min and re-equilibrated for 6 minutes. Mobile phase flow rate was 0.3 mL/min and the column was heated to 30 °C. The QTOF scanned at a range of 65 – 1100 m/z in MS-only mode at a scan rate of 4 spectra/s. Drying gas was 250 °C at 10 L/min, sheath gas was 350 °C at 10 L/min. The nebuliser was set at 45 psig. Capillary voltage was 2 000 V, no nozzle voltage was applied. MS/MS spectra were acquired during the equilibration runs using auto-MS/MS (data-dependent acquisition) at a scan rate of 8 spectra/s using a threshold of

minimal 2 000 counts and a maximum of 12 precursors per scan cycle. The quadrupole was set at a small width (1.3 amu) and the collision cell was set at 10, 20 and 40 V.

The TOF was tuned and calibrated using a reference mix. The LC-Q-TOF-MS was equilibrated using 15 injections of the QC-pool in *auto-MS/MS* mode. The injections of the samples were block-randomised and analysed in MS-only mode. Every 4 sample injections were followed by a QC- and a blanco-injection.

SI-5: parameters of the data preparation

Acquired data were imported to the MassHunter Qualitative software (Agilent Technologies, v 2.06.00) and converted to centroid m/z data and loaded in R. Features representing the ions of the extracted metabolites were searched using XCMS[5] using the centWave algorithm at a ppm threshold of 30, a signal to noise ratio of 6 and a noise-level of 1000. Features were aligned using the Obiwarp algorithm using local alignment and a response of 10 for the non-polar fraction and 20 for the polar fraction[6]. Features were grouped by density using with the following parameters: bw = 5, mzwid = 0.015, minfrac = 0.75, max 100. Missing peaks were re-extracted using the fillPeaks algorithm, noisy features were removed (RTwidth >100 or < 5, ppm.diff >50, gauss < 0.4)

The dataset was cleaned up using the MetaboMeeseeks package by removing isotopes (r > 0.8 of peaks with), features present in blank samples (signals with median abundance > 10 x highest blank in the non-polar fraction and >3 x highest blank in the polar fraction), a frequency filter (present in at least 80 % of samples within a group) and a variability filter (RSD <30 % & 40 % within at least one exposure group for the non-polar and polar fraction, respectively) reduced noisy features[7]. A principal component analysis (PCA) was performed and samples that defined PC 1 or PC 2 with a score difference >5 of the all other samples according to the skewed PC were considered as and removed for further analysis. After outlier removal, the filter process was re-iterated and samples were normalised using probabilistic quotient normalisation[8]. Missing values in extracted features were checked for randomness through a Fisher-test between the different exposure groups and missing values within features with a random missing value distribution (p>0.05) were imputed through k-nearest neighbour algorithm with 5 neighbours [8,9].

SI-6: Metabolite annotation

In MassHunter, the signals corresponding to potential markers of toxicity were selected, the complete result set was extracted and the Molecular Formula Generator (MFG) generated a list of possible chemical formulas. The identification was based on the m/z-value, the isotope pattern, the measured retention time and the fragmentation spectra acquired during the equilibration runs. The most plausible formulas were selected, based on the biochemical plausibility, the total matching score, the ppm-deviations (max. 10 ppm) and the fitting of the isotope pattern (max. 5 % deviation for the m+1 isotope and 1 % for the m+2 isotope)[10]. The measured m/z-values, combined with the MS/MS fragmentation spectra from the equilibration runs were chemically interpreted and compared to experimentally confirmed or *in silico* predicted reference spectra from the metabolite databases METLIN, LIPID MAPS, the Human Metabolome Database (HMDB) and/or ChemSpider[11–16].

The resulting levels of identification are based on the standardised reporting rules, published by the Chemical Analysis Working Group (CAWG) and the Metabolomics Standards Initiative (MSI) [17,18]. Briefly, identification can be related to five levels of confidence: level 5 being the lowest which only provides an m/z value. Level 4 is a low-confident annotation and provides a chemical formula. Level 3 has been divided into two categories: annotation based on mass spectrometric characteristics of the feature and retention times without (3B) and with fragmentation spectra (3A) and is related to at least a class-wise annotation. Level 2 identification is a hit confirmed with a reliable retention time and a confirmation of the fragmentation spectra with one of the database libraries. Level 1 identification is a complete similarity with an injected reference standard.

The identification of carnitine is used as an example for the identification process [19].

Feature m/z 162.1139 at a retention time of 8.7 was selected as a significant feature (corrected p-value < 0.05; RF VIM: 3.3, PLS VIP: 1.16, FC 0.8 & 0.7 for the first and second experiment, respectively). The extracted ion chromatogram in figure SI-14.A and the boxplots in SI-14.B confirm the results from the statistical test. Furthermore, the signal is higher than the blank, relating the signal to an endogenous metabolite from the culture.

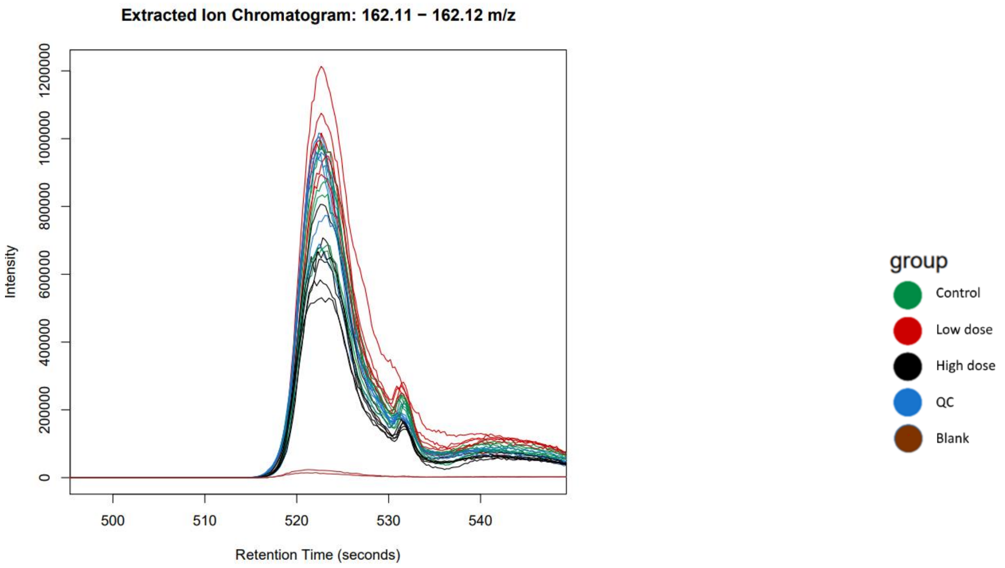

Fig. SI-6.A: Extracted ion chromatogram for all samples in experiment 1 for m/z 162.1139 at a retention time of 8.7. Samples of cultures exposed to the low dose have slightly higher signals in comparison to the negative control samples, samples of cultures exposed to the high dose have lower signals in comparison to the negative control culture. The signals are higher than the blank samples, indicating a true metabolic feature.

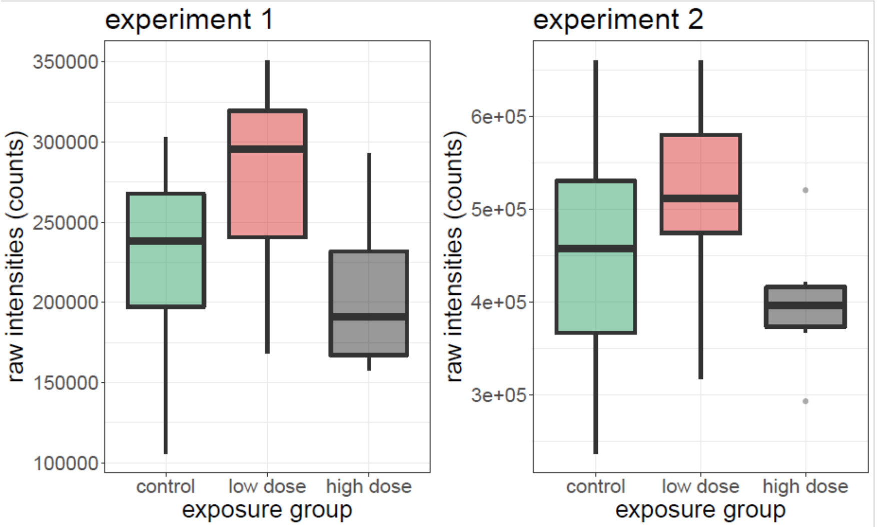

Fig. SI-6.B: Boxplots for m/z 162 at rt 8.7 min. The plots represent an initial upregulation of the metabolite, followed by a later downregulation, which is confirmed with a p-value <0.05.

Identification was performed using Mass Hunter qualitative analysis; the m/z value related to the molecular feature was grouped in a molecular feature with corresponding isotopes and additional adducts, such as m/z 184.095 and 200.068. Mass accuracy values were good (<1 ppm for the mono-isotopic value; <10 for the low-abundant isotopes) and the experimental isotope ratios match the theoretical values, confirming the formula of the feature.

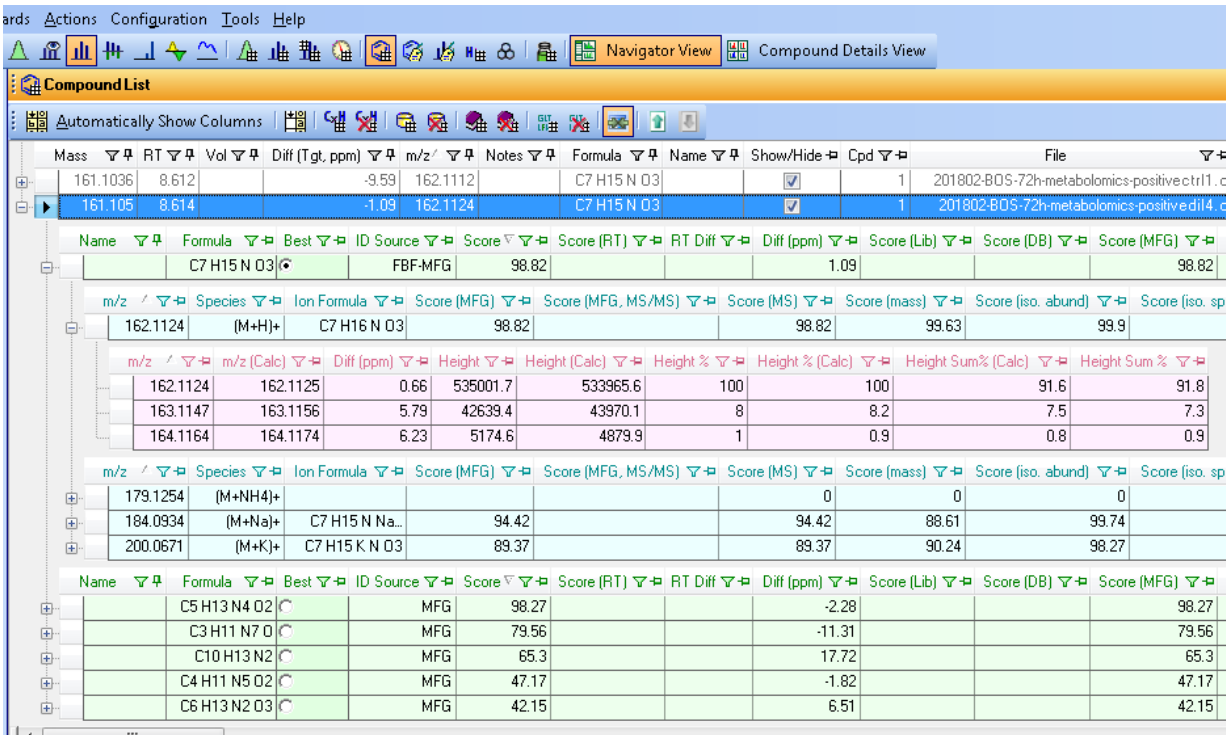

Fig. SI-6.C: Annotation of the molecular formula using the Agilent Molecular Formula Generator reveals a match for the proton, sodium and potassium adduct of a molecule with formula $C_7H_{15}NO_3$.

Fragments obtained during the equilibration runs of m/z = 162 at 8.7 minutes are shown in figure SI-14.D. The fragmentation pattern matches well with the experimentally acquired fragments of carnitine in the curated Metlin-library. Therefore, the substructures of the feature are related to carnitine. The combination of the molecular formula, an acceptable retention time and a matching fragmentation spectrum concluded the annotation of this feature as carnitine, with a confidence of level 2.

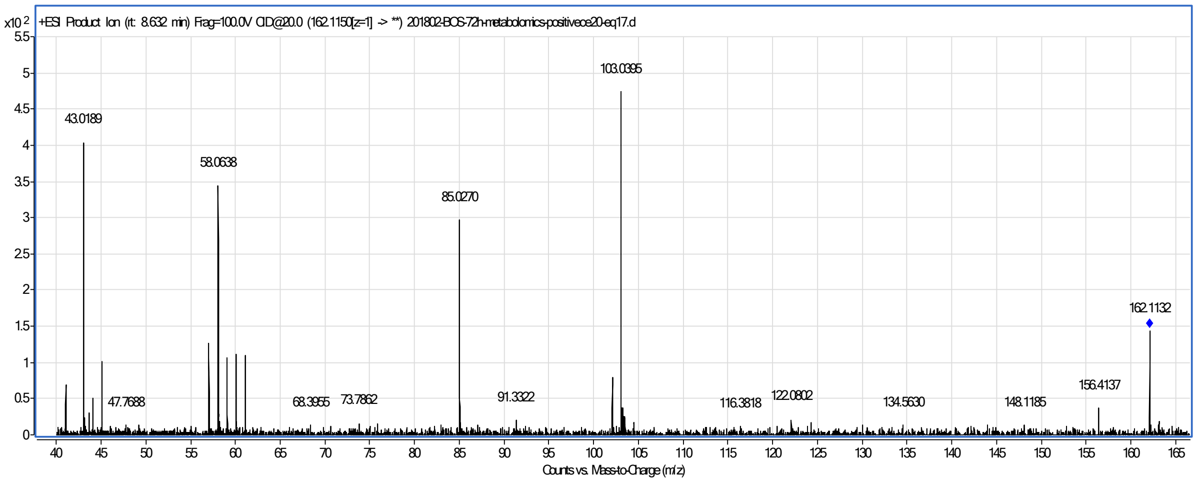

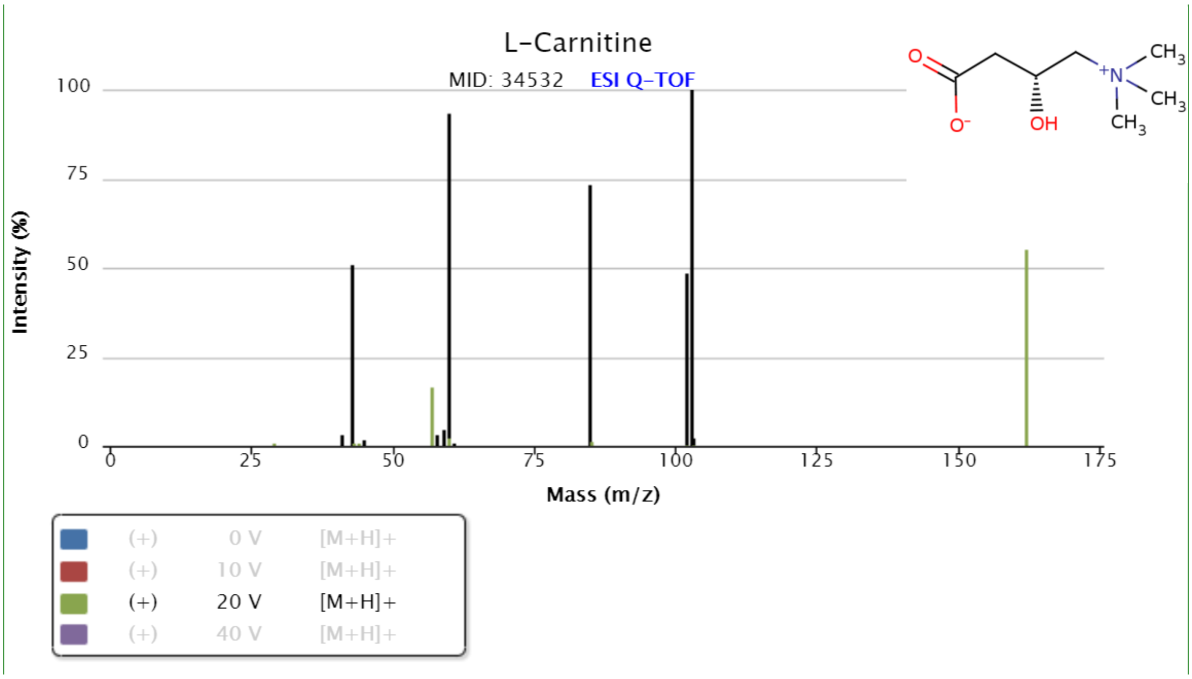

Fig. SI-6.D: MS/MS spectrum of the molecular feature (up), which matches with the MS/MS spectrum of L-carnitine in the METLIN database[14] (down).

SI-7: Quality control

Table SI-7.1: Samples which did not meet QC criteria. N/A: not applicable.

	Non-polar negative	Non-polar positive	Polar negative	Polar positive
72h-1	IC10-6, QC-1	QC-1/QC2	N/A	N/A
72h-2	N/A	N/A	N/A	N/A

Injections exp1-QC-1 and exp1-QC-2 failed in the non-polar fraction positive ionisation mode, reflected by the absence of internal standards. Figure SI-7.1 represents the PCA-plot that revealed outliers exp1-IC10-6 and exp1-QC-1

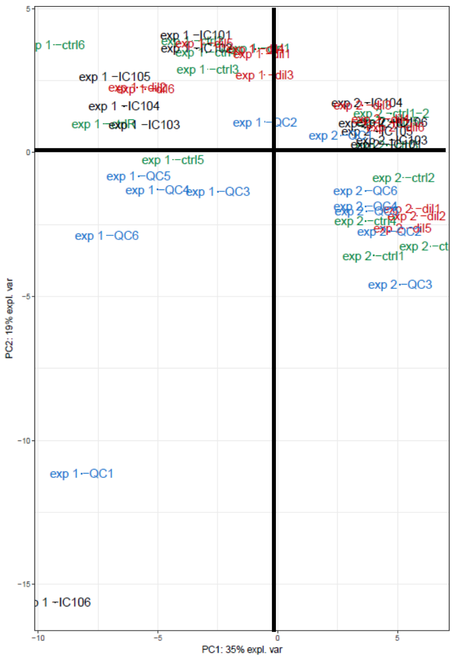

Figure SI-7.1: PCA reflecting the outlier position of QC-1 and IC10-6 for the dataset of the non-polar fraction in negative ionisation mode.

Table SI-7.2: Median relative standard deviations (mRSDs) for all subgroups in the experiments.

		QC	ctrl	Low-dose	High-dose
non-polar positive	72h-1	11.4	24.0	23.8	21.2
	72h-2	10.6	18.2	20.7	16.6
non-polar negative	72h-1	27.2	38.7	38.3	37.9
	72h-2	33.6	57.0	51.1	55.7
polar positive	72h-1	33.6	36.4	33.9	30.3
	72h-2	23.1	24.6	29.7	34.9
polar negative	72h-1	37.5	32.5	42.3	40.8
	72h-2	32.1	40.7	38.0	39.4

SI-8: Figures representing PCAs of the final datasets (green: negative control, red: low dose, black: high dose, blue: QC; circles: experiment 1 and triangles: experiment 2).

Figure SI-8A: PCA plots of the non-polar fraction in negative mode during the 72 h exposure showing PC1 vs PC2 (upper) and PC3 vs PC4 (lower). A broad clustering of the QC samples indicates the lower quality of the data-acquisition for experiment 2. There is strong overlapping of the different exposure groups in all principal components, indicating the variance is not related to exposure.

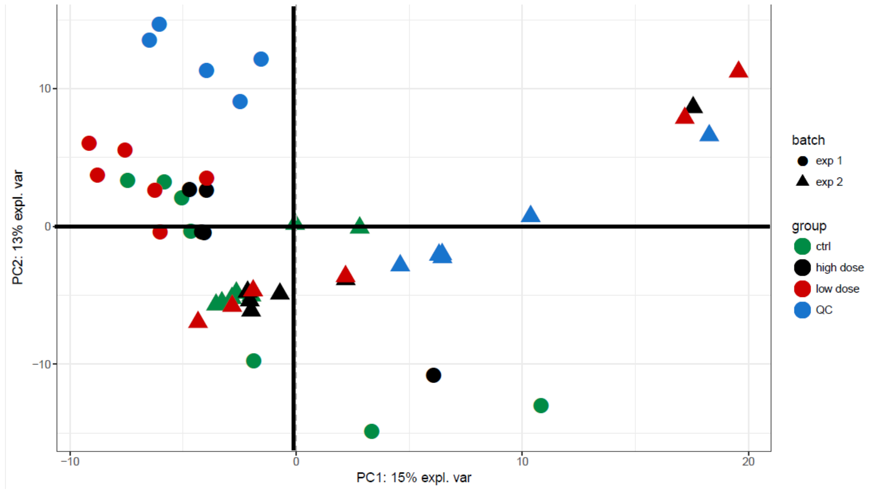

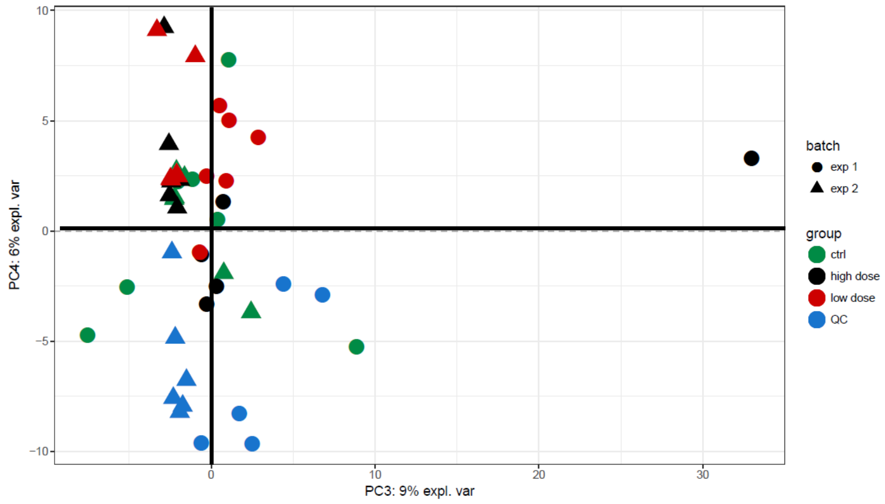

Figure SI-8B: PCA plots of the non-polar fraction in positive mode during the 72 h exposure showing PC1 vs PC2 (upper) and PC3 vs PC4 (lower). There is strong overlapping of the different exposure groups in all principal components, indicating the variance is not related to exposure. Only a slight trend is visible between the negative control group and the group exposed to the higher dose.

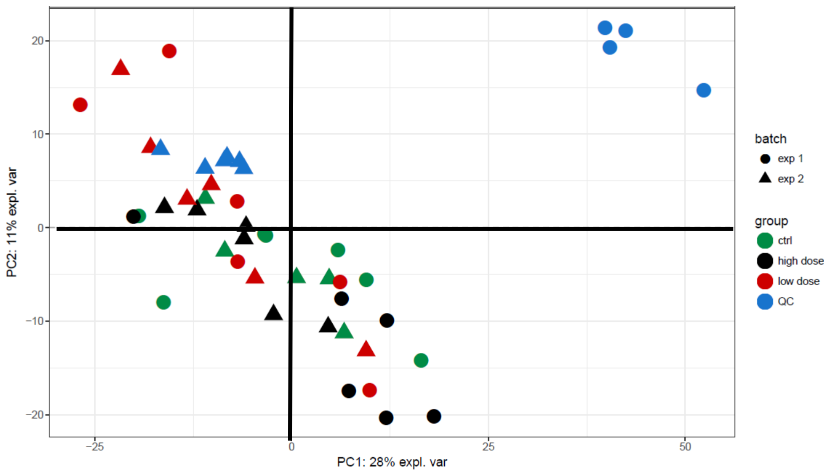

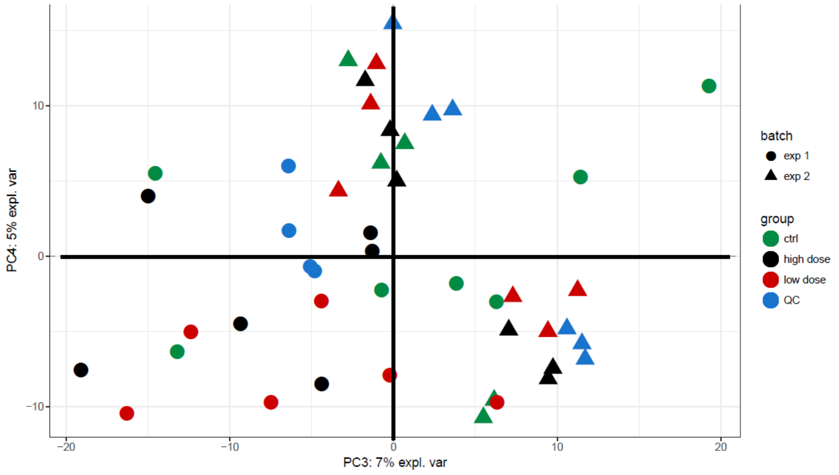

Figure SI-8C: PCA plots of the polar fraction in negative mode during the 72 h exposure PC1 vs PC2 (upper) and PC3 vs PC4 (lower). Discriminatory trends between the low dose and the negative control group are not represented in the major four components. The less dense projection of the QC samples indicates the introduction of variance during data-acquisition and processing.

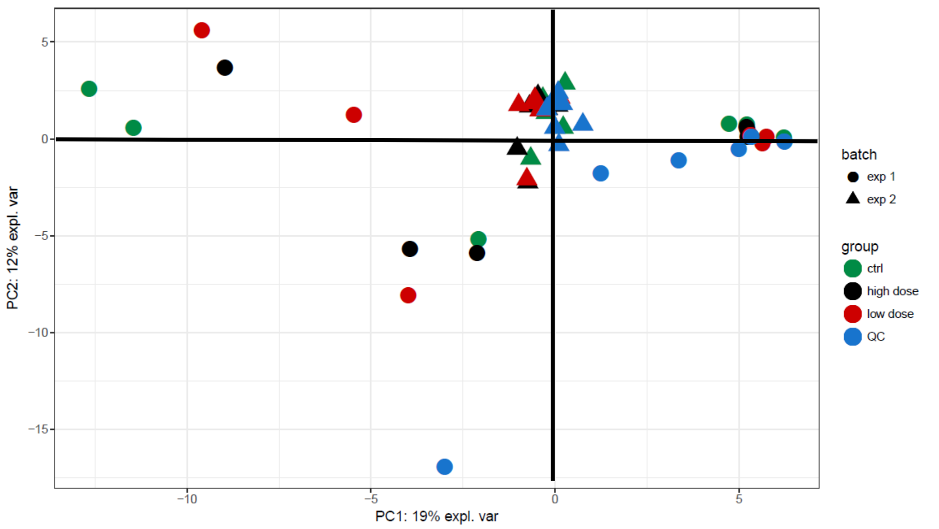

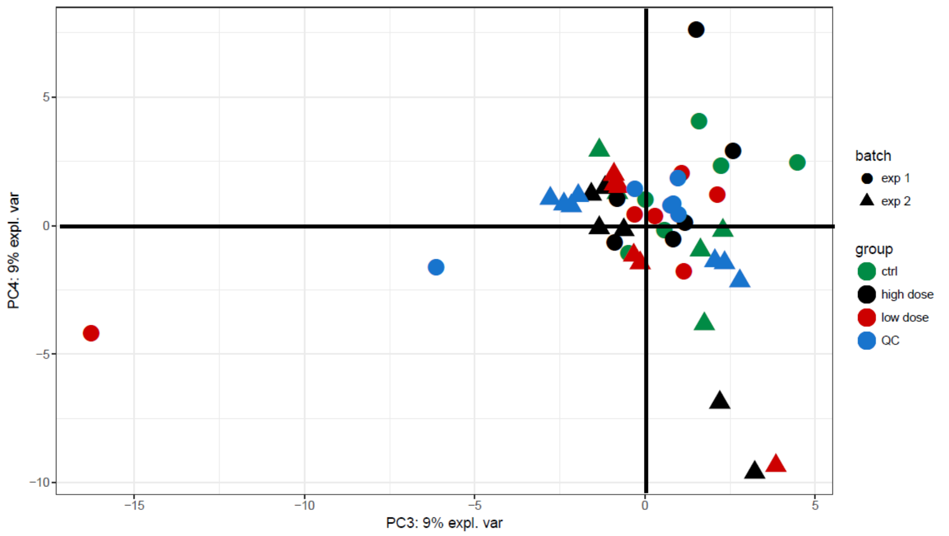

Figure SI-8D: PCA plots of the polar fraction in positive mode during the 72 h exposure showing PC1 vs PC2 (upper) and PC3 vs PC4 (lower). The less dense projection of the QC samples indicates the introduction of variance during data-acquisition and processing. There is strong overlapping of the different exposure groups in all principal components, indicating the variance is not related to exposure.

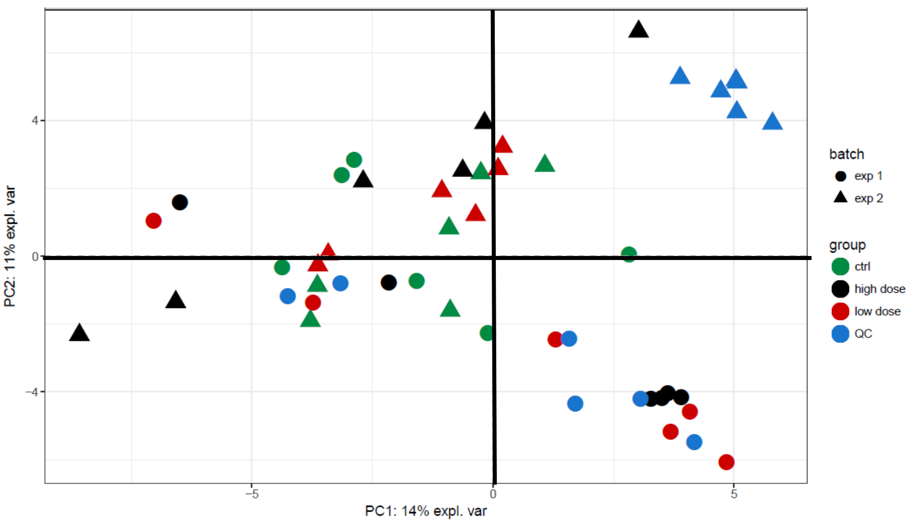

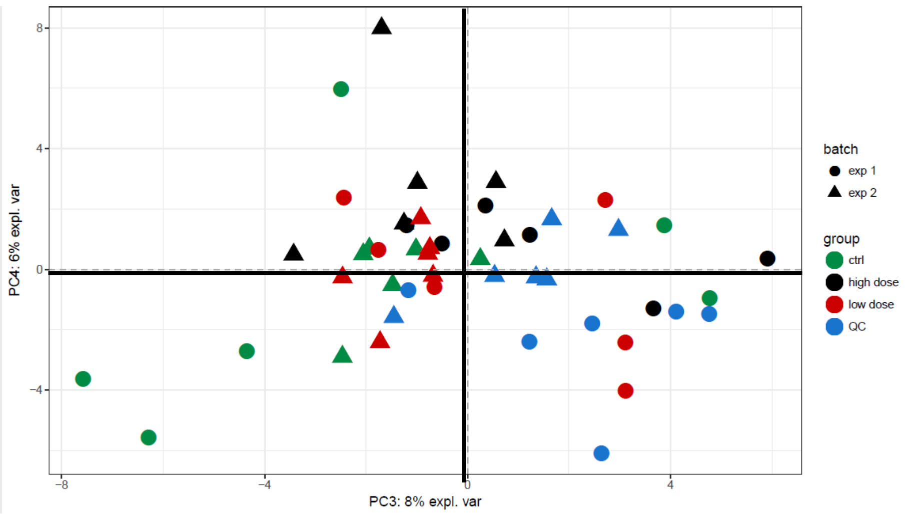

Table SI-9: Metabolites identified as potential metabolites of interest for all exposure models. The table is provided in an additional excel file.

SI-10: Boxplots of metabolites mentioned in table SI-11 in both experimental replicates. Feature ID refers to the features in Table SI-11. The boxplots are provided in an additional pdf-file.

References

1. Borenfreund, E.; Puerner, J. A simple quantitative procedure using monolayer cultures for cytotoxicity assays (HTD/NR90). *J. Tissue Cult. Methods* **1984**, *9*, 7–9.

2. Ates, G.; Vanhaecke, T.; Rogiers, V.; Rodrigues, R.M. Assaying cellular viability using the Neutral Red Uptake assay. Methods in Molecular Biology. In *Methods in Molecular Biology*; 2017; pp. 19–26.

3. Wu, H.; Southam, A.D.; Hines, A.; Viant, M.R. High-throughput tissue extraction protocol for NMR- and MS-based metabolomics. *Anal Biochem* **2008**, *372*, 204–212.

4. Cuykx, M.; Mortelé, O.; Rodrigues, R.M.R.M.; Vanhaecke, T.; Covaci, A. Optimisation of in vitro sample preparation for LC-MS metabolomics applications on HepaRG cell cultures. *Anal. Methods* **2017**, *9*, 3704–3712.

5. Smith, C.A.; Want, E.J.; Maille, G.O.; Abagyan, R.; Siuzdak, G. XCMS : Processing mass spectrometry data for metabolite profiling using nonlinear peak alignment , matching , and identification. *ACS Publ.* **2006**, *78*, 779–787.

6. Prince, J.T.; Marcotte, E.M. Chromatographic alignment of ESI-LC-MS proteomics data sets by ordered bijective interpolated warping. *Anal. Chem.* **2006**, *78*, 6140–6152.

7. Beirnaert, C.; Cuykx, M.; Bijtebier, S. MetaboMeeseeks: Helper functions for metabolomics analysis. *R Packag.* **2019**, version 0.1.10044.

8. Dieterle, F.; Ross, A.; Schlotterbeck, G.; Senn, H. Probabilistic quotient normalization as robust method to account for dilution of complex biological mixtures. Application in1H NMR metabonomics. *Anal. Chem.* **2006**, *78*, 4281–4290.

9. Di Guida, R.; Engel, J.; Allwood, J.W.; Weber, R.J.M.; Jones, M.R.; Sommer, U.; Viant, M.R.; Dunn, W.B. Non-targeted UHPLC-MS metabolomic data processing methods: a comparative investigation of normalisation, missing value imputation, transformation and scaling. *Metabolomics* **2016**, *12*, 1–14.

10. Kind, T.; Fiehn, O. Seven Golden Rules for heuristic filtering of molecular formulas obtained by accurate mass spectrometry. *BMC Bioinformatics* **2007**, *8*, 105.

11. Sud, M.; Fahy, E.; Cotter, D.; Brown, A.; Dennis, E.; Glass, C.; Murphy, R.; Raetz, C.; Russell, D.; Subramaniam, S. LMSD: LIPID MAPS Structure Database. *Nucleic Acids Res.* **2007**, *35*, D527-532.

12. Pence, H.; Williams, A. ChemSpider: An Online Chemical Information Resource. *J. Chem. Educ.* **2010**, *87*, 1123–1124.

13. Godzien, J.; Ciborowski, M.; Martínez-Alcázar, M.P.; Samczuk, P.; Kretowski, A.; Barbas, C. Rapid and Reliable Identification of Phospholipids for Untargeted Metabolomics with LC-ESI-QTOF-MS/MS. *J. Proteome Res.* **2015**, *14*, 3204–3216.

14. Smith, C.A.; O'Maille, G.; Want, E.J.; Qin, C.; Trauger, S.A.; Brandon, T.R.; Custodio, D.E.; Abagyan, R.; Siuzdak, G. METLIN - A metabolite mass spectral database. *Ther. Drug Monit.* **2005**, *27*, 747–751.

15. Fahy, E.; Sud, M.; Cotter, D.; Subramaniam, S. LIPID MAPS online tools for lipid research. *Nucleic Acids Res* **2007**, *35*, W606-12.

16. Wishart, D.S.; Jewison, T.; Guo, A.C.; Wilson, M.; Knox, C.; Liu, Y.F.; Djoumbou, Y.; Mandal, R.; Aziat, F.; Dong, E.; et al. HMDB 3.0-The Human Metabolome Database in 2013. *Nucleic Acids Res* **2013**, *41*, D801–D807.

17. Schymanski, E.L.; Jeon, J.; Gulde, R.; Fenner, K.; Ruff, M.; Singer, H.P.; Hollender, J. Identifying small molecules via high resolution mass spectrometry: Communicating confidence. *Environ. Sci. Technol.* **2014**, *48*, 2097–2098.

18. Sumner, L.W.; Amberg, A.; Barrett, D.; Beale, M.H.; Beger, R.; Daykin, C.A.; Fan, T.W.M.; Fiehn, O.; Goodacre, R.; Griffin, J.L.; et al. Proposed minimum reporting standards for chemical analysis: Chemical Analysis Working Group (CAWG) Metabolomics Standards Initiative (MSI). *Metabolomics* **2007**, *3*, 211–221.

19. Cuykx, M.; Claes, L.; Rodrigues, R.M.; Vanhaecke, T.; Covaci, A. Metabolomics profiling of steatosis progression in HepaRG®cells using sodium valproate. *Toxicol. Lett.* **2018**, *286*, 22–30.
